# Preterm delivery rate in China: a systematic review and meta-analysis

**DOI:** 10.1186/s12884-022-04713-z

**Published:** 2022-05-02

**Authors:** Qinfeng Song, Junxi Chen, Yubo Zhou, Zhiwen Li, Hongtian Li, Jianmeng Liu

**Affiliations:** 1 Institute of Reproductive and Child Health, National Health Commission Key Laboratory of Reproductive Health, Peking University Health Science Center, Beijing, 100191 China; 2 Department of Epidemiology and Biostatistics, School of Public Health, Peking University Health Science Center, Beijing, 100191 China

**Keywords:** Preterm delivery, Rate, China, Meta-analysis

## Abstract

**Background:**

Preterm delivery rate is a crucial public health indicator, yet reliable statistic is currently not available in China. In this systematic review and meta-analysis, we aimed to review studies on preterm delivery rate in China, explore sources of heterogeneity, and estimate the preterm delivery rate in China.

**Methods:**

Published studies on preterm delivery rate in China since 2010 were electronically searched from PubMed, Embase, Web of Science, China National Knowledge Infrastructure, China Science and Technology Journal Database, and Wanfang Database, and complemented by manual search. Study selection, data extraction, and quality and bias assessment (using the Joanna Briggs Institute Critical Appraisal Checklist) were conducted by two reviewers independently. Random-effects meta-analysis was performed to estimate the pooled preterm delivery rate, and prespecified stratified analysis was conducted to explore sources of heterogeneity.

**Results:**

The database search returned 4494 articles and manual search identified 10 additional studies. In total, 162 studies were eligible, of which 124 were hospital-based and 38 population-based. The pooled preterm delivery rate of hospital-based studies (7.2%; 95% CI: 6.9% to 7.6%) was significantly higher than that of population-based studies (4.9%; 95% CI: 4.5% to 5.4%) (*P* for subgroup difference < 0.001). Among population-based studies, the rate tended to differ by geography (*P* for subgroup difference = 0.07): 5.3% for Eastern, 4.6% for Central, and 3.8% for Western.

**Conclusions:**

According to population-based studies, the preterm delivery rate in China is around 5%. This rate is substantially lower than estimates from hospital-based studies or estimates from a combination of both hospital-based and population-based studies as having been done in previous studies.

**Supplementary Information:**

The online version contains supplementary material available at 10.1186/s12884-022-04713-z.

## Background

Preterm delivery is widely defined as a delivery at less than 37 gestational weeks [1]. This upper boundary is rather consistent across nations, while the lower boundary varies, e.g. 20 weeks in America, 22 weeks in Europe, and 28 weeks in China [2–4]. As a multifactorial syndrome, preterm delivery is one of the most common adverse pregnancy outcomes, with a globally-estimated rate of 10.6% in 2014 [1]. It is the leading cause of death among children under five worldwide [1], and is also the leading cause of neonatal death in China [5]. Moreover, schooling difficulties or behavioral problems are more likely among prematurely-delivered children, and these difficulties may even persist into adolescence [6]. Therefore, the preterm delivery rate is a widely-used indicator for monitoring maternal and child health. Reliable national statistics on premature births, however, are currently not available in the literature for many countries including China.

China accounts for more than 10% of births worldwide [7]. As a result, reliable estimate of preterm delivery rate for China is crucial to both domestic and international disease burden estimation. The rate among live births in China was estimated to be 6.9% in 2014 according to a World Health Organization (WHO) modelling study [1], and 7.0% in 2015–2016 according to a subsequent meta-analysis [8]. The WHO study included a total of 102 data points of China identified from seven English databases and six most highly cited medical journals in one Chinese database, while the subsequent meta-analysis included a total of 187 data points identified from the same sources as the WHO study.

These two studies, however, did not differentiate between population- and hospital-based studies, or between studies from different levels of hospitals. This might have led to unreasonable estimates in the presence of high-risk referral of maternal management in China and the overrepresenting of studies from tertiary hospitals [9], since studies with different data sources represent different target population. Tertiary hospitals in China, with a stronger capacity for clinical service, usually serve as referral centers and admit pregnancies with increased risk of preterm delivery. For example, in one study including 67 tertiary hospitals and 22 secondary hospitals, the average preterm delivery rate of tertiary hospitals was almost twice that for secondary hospitals (10.0% *vs*5.4%) [10]. Tertiary hospitals in China also have relatively strong research capacities, so studies from tertiary hospitals on preterm delivery rates may be highly overrepresented than those from secondary hospitals in the literature. Therefore, combining all the studies without differentiating data sources of studies might have overrepresented studies from tertiary hospitals and overestimated the burden of preterm deliveries.

In this systematic review and meta-analysis, we aimed to estimate the national preterm delivery rate in China with careful consideration of data sources (i.e., population-based studies versus hospital-based studies), and to assess other factors that may influence the reported preterm delivery rates in the literature.

## Methods

### Search strategy and selection criteria

In this systematic review and meta-analysis, an electronic literature search was executed by two reviewers (QFS and JXC) from the following electronic databases: PubMed, Embase, Web of Science, China National Knowledge Infrastructure (CNKI), China Science and Technology Journal Database (VIP) and Wanfang Data. A combination of key terms (‘Preterm Delivery’, ‘China’, ‘Rate’, and their synonyms) was adopted to identify potentially eligible studies published in English or Chinese between January 1, 2010 and July 31, 2019. Studies prior to 2010 were not included, as the long-term temporal trends in the preterm delivery rate was beyond the scope of this study. The detailed search strategy is provided in eText1. The electronic search was complemented by manual search of the reference list of the recent meta-analysis [8]. Of note, this recent meta-analysis was an updated analysis of and used the same search strategy as the WHO study [1], so all the literatures involved in the WHO study have been included in this recent meta-analysis. Therefore, we did not do further manual search of the reference list of the WHO study.

Titles and abstracts of searched studies were independently screened for relevance and appropriateness of inclusion by two reviewers (QFS and JXC). Prior to review of the full-texts, the two reviewers jointly checked the studies that were only retained by one of them. To be eligible for the systematic review, the study needed to report the preterm delivery rate in China or relevant data from which it can be calculated. Studies were excluded if: 1) The data were generated before January 1, 2010, or the data were mostly generated before 2010 and the preterm delivery rate in 2010 and subsequent years could not be obtained; 2) The overall preterm delivery rate was not available (e.g., non-human studies, case–control studies, or studies only focusing on iatrogenic or spontaneous preterm delivery); 3) The study dealt with a special population (e.g., only assisted reproduction, only twin pregnancies, only multiparas, or only those in particular occupations), considering that combining studies from these typical populations with those from more general populations may lead to unexplainable pooled results; 4) Experimental studies; 5) Sample size ≤ 500; 6) Review, commentary, expert opinion, case report, etc.; 7) Duplicate reports from the same study or studies originating from the same population; 8) Studies based on routine statistical reports of aggregated data, which might suffer from underreporting bias [11]. As for the meta-analysis, studies without reporting total participants and preterm delivery cases were further excluded. Full-text review was also processed independently by two reviewers (QFS and JXC), and confirmed by a third reviewer (HTL) in case of any discrepancies. This study followed the PRISMA checklist (eTable1) and was registered on the PROSPERO (registration number: CRD42020145415).

### Data analysis

The data were collected using a standardized data extraction form. The extracted information included publication year, first author, language, study year, study setting, data source (population- or hospital-based), hospital level, singleton only (yes or no), lower and upper gestational week boundaries of preterm delivery definition, live birth only (yes or no), unit of analysis (woman or neonate), and number of total participants and preterm delivery cases (the preterm delivery rate was extracted when these numbers were not available). For studies initiated before 2010, only data on 2010 and subsequent years were extracted. For studies lasting for more than one year, the study year was recorded as the median time of the study period. For studies involving multiple settings, setting-specific data was extracted, if possible. Study settings were initially recorded as the provinces where the studies occurred, which were ultimately grouped into four traditional geographic regions: Eastern, Central, Western and Northeast China [12]. Studies that included more than one geographic region were not included in corresponding stratified analysis, if region-specific data were not available. Population-based studies refer to those covering all deliveries of a region or a sample of deliveries of a region. Hospital-based studies refer to those on the basis of medical records from a single hospital or from a group of non-randomly selected hospitals of a region. The level of hospitals was determined by referring to the classification information in 2020 from the National Health Commission of the People’s Republic of China [13]. Given the observational nature of this review, the Joanna Briggs Institute (JBI) Critical Appraisal Checklist for Prevalence Studies (2017) was used to assess the quality of the studies and determine potential bias [14]. This checklist involves a total of 9 items, and the answer options are set to “yes”, “no”, “unclear”, and “not applicable”. Data extraction and quality and bias assessment were done independently by two reviewers (QFS and JXC), and disagreements were resolved by discussion with HTL.

Heterogeneity across studies was assessed with *I*^*2*^ statistic and *P* values of Cochrane Q statistic. An a priori decision was made to use the random-effects model for all the analyses in anticipation of substantial heterogeneity between studies. After improving the normality of rates by logarithmic transformation, the log rate was pooled by the inverse variance method. The pooled rate was presented as percentages accompanying with 95% confidence intervals (CIs). Forest plots were used to graphically represent the data. Several sets of prespecified stratified analyses were conducted to explore potential sources of heterogeneity. Sensitivity analysis was carried out to explore the robustness of meta-analysis results by sequentially excluding each individual study. Publication bias was firstly examined by visual inspection of the funnel plots and then tested using the Peters’ test in anticipation of substantial heterogeneity between studies [15, 16]. Management of literature was accomplished using Endnote, version X9 (Thomson ResearchSoft, Connecticut, United States). All analyses were carried out using R software, version 4.0.3 (The R Foundation for Statistical Computing, Vienna, Austria), and meta-analysis was performed with the “meta” package [17]. Two-tailed *P* < 0.05 was considered statistically significant.

## Results

**Fig. 1:**
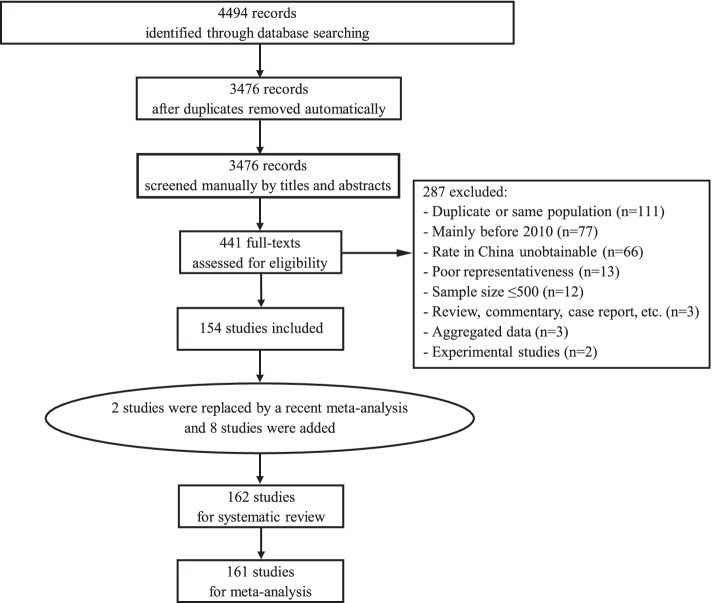
Study section

The database search returned a total of 4494 articles, of which 3476 were identified as non-duplicated records by Endnote. Among them, 441 were kept after initial screening of titles and abstracts. After review of the full-text articles, 154 met all criteria for inclusion. Additionally, manual search of the reference list of the recent meta-analysis identified 10 additional studies, and two of them replaced two out of the 154 studies for a better coverage of the same population (Fig. 1). A list of the 162 studies included in this systematic review is provided in the supplementary (eText2).

Of these studies, 68 were in English (42.0%) and 94 (58.0%) in Chinese. Population-based studies accounted for 23.5% (38/162), among which 25 were based on the whole population of a region and 13 were based on a sample of regional population. Hospital-based studies accounted for up to 76.5% (124/162), with the number of studies from tertiary hospitals almost 5 times that of secondary hospitals (69 *vs* 14; the remaining 41 studies simultaneously involved both tertiary and secondary hospitals). Among the 162 studies eligible for inclusion, 75 (46.3%) only included singleton births; 111 (68.5%) only included live births; 109 (67.3%) used woman as the unit of analysis. In terms of the definition of preterm delivery, 40.7% (66/162) of the studies used a lower boundary of 28 weeks, 19.1% (31/162) used a lower boundary of ≤ 27 weeks, and 40.1% (65/162) did not clarify the lower gestational week boundary. The median years in which data were collected ranged from 2010 to 2017. The settings of studies covered 30 out of the 31 provinces, autonomous regions, and municipalities in Chinese mainland (only not involving Qinghai Province). Overall, there were 190 region-specific preterm delivery rate records extracted from the 162 studies and 50.0% (95/190) of them were from Eastern China. The preterm delivery rates ranged from 1.6% to 29.0%. One eligible population-based study was not included in the meta-analysis because the numbers of total participants and preterm delivery cases of it were unobtainable. For the remaining 161 studies, the sample sizes ranged from 592 to 4,832,887. More details on the characteristics of the included studies are shown in eTable2.

The average consistency rate in 9 items of the JBI checklist between two assessors was 85.5%. The percentage of studies that were evaluated as “yes” exceeded 75.0% in 8 out of 9 items, such as appropriate sampling method, adequate sample size, and detailed description of the subject and setting (eTable3). However, 71.0% (115/162) of the studies lacked an appropriate sample frame to address the target population, in particular hospital-based studies, indicating a likelihood of presence of selection bias in these studies (eTable3).

**Fig. 2:**
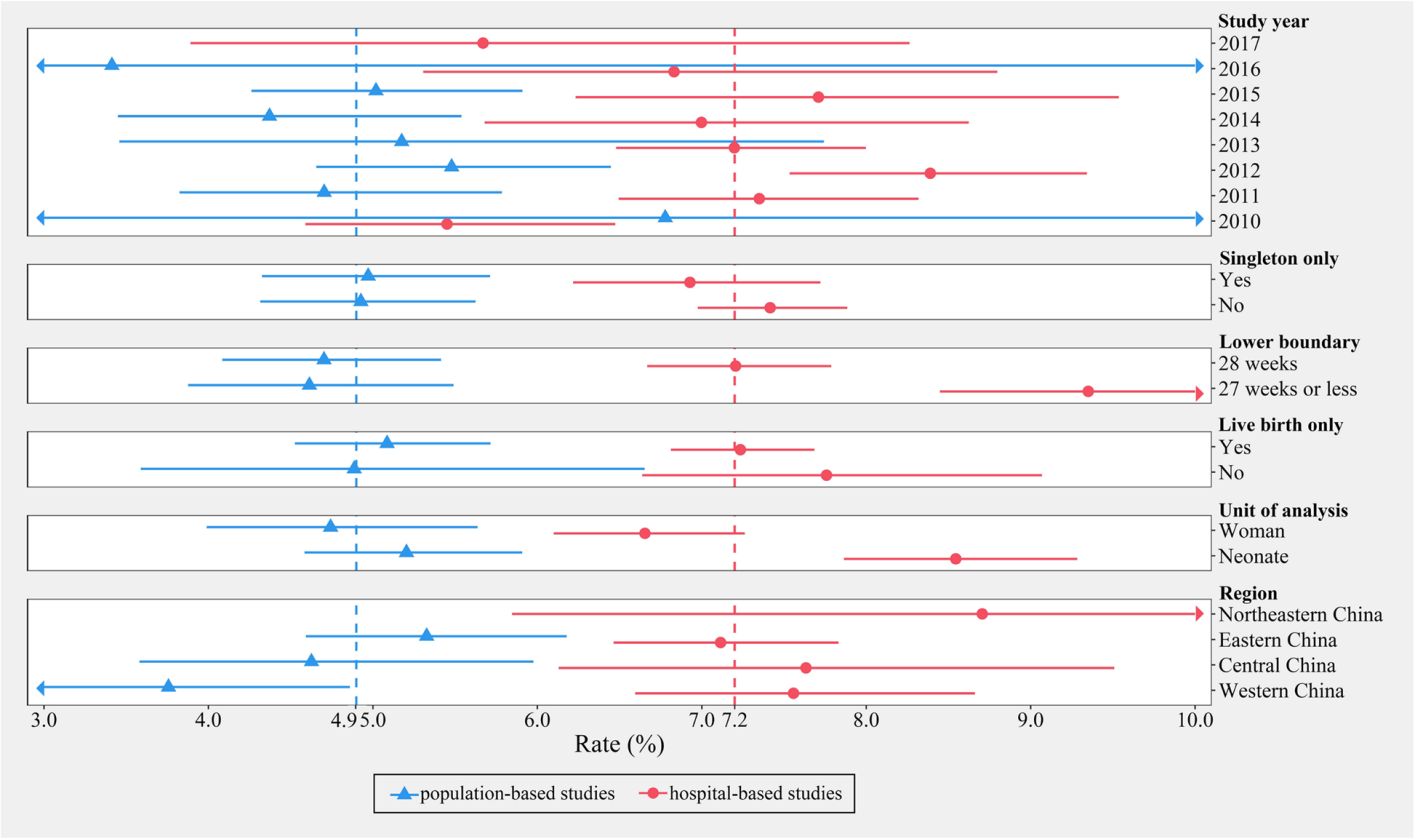
Stratified analyses within population-based and hospital-based studies

As anticipated, there was substantial statistical heterogeneity across the 161 studies (*I*^*2*^ = 99.8%) (eFigure1). Given the extremely large and subgroup-consistent difference in the preterm delivery rates between population- and hospital-based studies (Fig. 2), further analyses were separately performed for studies using different data sources.

**Fig. 3:**
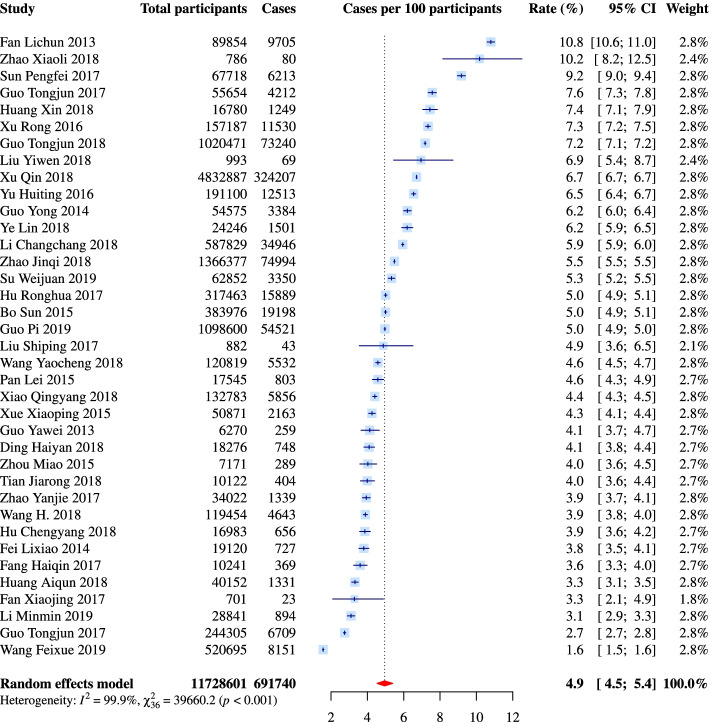
Forest plot of population-based studies

**Fig. 4:**
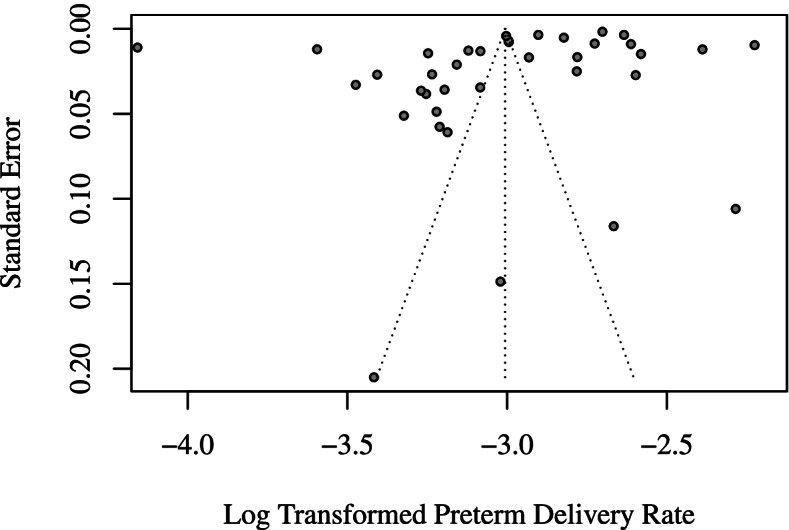
Publication bias of population-based studies

The 37 population-based studies involved a total of 11,728,601 participants, which resulted in a pooled preterm delivery rate of 4.9% (95% CI: 4.5% to 5.4%; *I*^*2*^ = 99.9%) (Fig. 3). Among these studies, 34 collected data from regional electronic medical databases (e.g. Birth Certificate System, Maternal and Child Health Care Network) that gathered information on deliveries occurred in hospitals. Sensitivity analysis showed that none of these individual studies significantly influenced the pooled rate (eFigure2). The CIs for rates of four small-scale studies were wide, but their point estimates were evenly distributed across the rate range of population-based studies (Fig. 3). The pooled analysis of studies with sample ≥ 2000 (*n* = 33), with a better identified sample coverage (*n* = 27), and with a response rate ≥ 85% (*n* = 33) generated almost identical rates: 4.9%, 5.0%, and 4.9%, respectively (eTable4). In stratified analyses, the pooled rates of population-based studies were comparable across strata defined by most study characteristics, including the language of publication (5.1% for studies published in English and 4.8% for studies published in Chinese; *P* for subgroup difference = 0.62), while there was a borderline significant difference among studies from different geographic regions (*P* = 0.07) (eTable4). The pooled rate of studies from Eastern China was the highest (5.3%; 95% CI: 4.6% to 6.2%), followed by that of Central (4.6%; 95% CI: 3.6% to 6.0%) and Western (3.8%; 95% CI: 2.9% to 4.9%) China (eTable4). No appreciable difference in the pooled rates across study years was observed (eTable4). The funnel plot of the 37 population-based studies was roughly symmetrical according to visual and intuitive judgment (Fig. 4), and Peters’ test also indicated no evidence of publication bias (*P* = 0.80).

The 124 hospital-based studies involved a total of 6,171,419 participants, which resulted in a pooled preterm delivery rate of 7.2% (95% CI: 6.9% to 7.6%; *I*^*2*^ = 99.5%) (eTable5). Significant differences in pooled preterm delivery rates were found in strata defined by study year, the lower boundary, the unit of analysis, and the level of hospitals (eTable5). A higher pooled rate was observed in studies using ≤ 27 weeks of gestation as the lower boundary to define preterm delivery, those using the neonate as the unit of analysis, and those from tertiary hospitals, in comparison to their counterpart subgroup. Most striking is the rate of tertiary hospitals, up to 60% higher than that of secondary hospitals (8.0% *vs* 4.9%). In the presence of poor representativeness of hospital-based studies, we did not attempt to assess the publication bias of these studies.

## Discussion

To our knowledge, this is the first study that took data sources into account when systematically reviewing and summarizing studies on the preterm delivery rate in China. As anticipated, the overall pooled rate of hospital-based studies was substantially higher than that of population-based studies, with a difference up to 2.3 percentage points. The pooled rate of population-based studies was 4.9% (95% CI: 4.5% to 5.4%), indicating that the rate of preterm delivery in China was around 5.0%.

In the sensitivity analysis, the pooled rate of population-based studies varied narrowly from 4.8% to 5.1% with each study sequentially excluded, and the pooled results of several major subgroups (i.e., studies with larger sample size, better coverage, and higher response rate) were almost identical to the overall pooled rate, indicating its robustness. Of note, one population-based study included in the systematic review but not in the meta-analysis was conducted based on sentinel-surveillance data collected from 16 counties in Hubei province during 2001–2012, which reported a preterm delivery rate of 10.5% in 2012 (without reporting the specific number of births and preterm delivery cases in 2012) [18]. Although to which extent it can represent the entire population of Hubei province is unclear, another population-based study involving whole population in Wuhan, the provincial capital of Hubei, only reported a preterm delivery rate of 5.0% [19]. To be reassured, we made a rough estimation of the number of live births in 2012 for that sentinel-surveillance study by dividing the reported total live births during 2001–2012 by 12, and found that the pooled rate only slightly increased to 5.0% (95% CI: 4.6% to 5.5%) after adding that study to the meta-analysis. In addition, two population-based studies included in our study reported a preterm delivery rate less than 3%, a level that was considered biologically implausible in the WHO study [1]. If omitting them, the pooled rate would be 5.2% (95% CI: 4.9% to 5.6%).

The included population-based studies had a broad geographic coverage, where more than 70% of total births in China occurred [20]. In our study, the region-specific pooled rate tended to be higher in Eastern China as compared with Central and Western China. Eastern is the most developed area with the lowest elevation in China, and many factors including the aggregation of high-level hospitals, increased psychological distress during pregnancy, increased atmospheric pressure, and ambient temperature exposures, may partially account for its higher rate [21–23]. In spite of the geographic heterogeneity, our post-hoc analysis interestingly showed that the weighted national preterm delivery rate, calculated by weighting the province-level pooled preterm rate in our study with total live births in each province during 2010–2018 (eTable6), was 4.8% (95% CI: 4.0% to 5.9%), almost the same as the overall pooled rate.

The preterm delivery rate in China has been estimated to be 7.1% in an earlier WHO study [24], 6.9% in a recent WHO study [1], and 7.0% in a recent meta-analysis [8]. Notably, neither the WHO studies nor the meta-analysis differentiated between population- and hospital-based studies. In our study, a combination of both population- and hospital-based studies would lead to a pooled rate of 6.6% (95% CI: 6.3% to 6.9%) under the random-effects model. Such a pooled rate, however, would probably overestimate the burden of preterm deliveries. To be specific, the numbers of tertiary hospitals and their inpatients in China are merely 0.3 and 1.3 times that of secondary hospitals [25], but in our study the numbers of studies and participants from tertiary hospitals were up to 4.9 and 4.1 times that of secondary hospitals, respectively. Obviously, hospital-based studies on preterm delivery rate in the literature highly overrepresented tertiary hospitals. Due to this stubborn bias and the inherent difference in preterm delivery rates between tertiary and secondary hospitals (8.0% *vs* 4.9% in our study; 10.0% *vs* 5.4% in another study [10]) caused by high-risk referral, pooling both population-based and hospital-based studies will probably overestimate the actual rate of preterm delivery in China. One quantizable impact of it is that the annual number of preterm deliveries in China would largely reduce from more than 1.15 million in the recent WHO study to about 0.8 million estimated based on our pooled rate [1]. The decrease of nearly 30% of the rate (from 6.9% to 4.9%) made the rank of China dropped from 160 to 180 among 183 countries in the report [1].

Preterm delivery rate is a crucial public health indicator in maternal, newborn, and child health surveillance. After the announcement of China’s universal three-child policy [26], the demand of high-risk referral to tertiary hospitals will likely increase due to the anticipated increase in extremely advanced-aged pregnant women [27], which in turn may lead to an increase in preterm delivery rates in these hospitals. In the meanwhile, the recent decline in fertility in China may be associated with changes in preterm delivery rate [28], suggesting a need for monitoring of preterm deliveries in the near future. From a global perspective, in settings where population-based estimates on preterm delivery rate or other similar health indicators (such as the rate of low birth weight) are currently not available, data source should be taken seriously when conducting a pooling study, especially in settings where high-risk referral of patients exists.

Within hospital-based studies, we observed higher pooled rates for studies using ≤ 27 weeks of gestation as the lower boundary and for those using the neonate as the unit of analysis. These can be explained, respectively, by adding the same number to both the numerator and denominator when calculating the preterm delivery rate and by an increased risk of preterm delivery in multiple pregnant women [29]. Interestingly, the pooled rate of population-based studies using ≤ 27 weeks of gestation as the lower boundary of preterm delivery was not higher than that of studies using 28 weeks of gestation as the boundary. No matter whether the small group of extremely preterm deliveries will result in an appreciable rise in the overall preterm delivery rate, their growth and development should be closely monitored on account of the increased risk of adverse outcomes [30].

One advantage of this meta-analysis is that the studies were pooled with careful consideration of data source. Focusing on population-based studies dominantly based on electronic medical databases, we generated an estimate of preterm delivery rate in China using the random-effects model. Besides, we also made a comparison in pooled rates between hospital-based studies and population-based studies, which confirmed our speculation that the pooled rate of hospital-based studies was substantially higher than that of population-based studies. Considering that a portion of publications may appear in Chinese domestic journals, we simultaneously included studies published in English and Chinese, and were reassured by the consistent language-specific pooled results. In addition to the primary analysis, we also explored the potential impacts of some typical characteristics on the pooled preterm delivery rates, which may facilitate future study design and interpretation regarding preterm delivery rates in China and abroad.

Our study has several limitations. We used the relative new hospital level classification information to define the level of hospitals, which may lead to an underestimation of the preterm delivery rate in tertiary hospitals and overestimation of that in secondary hospitals, since a portion of hospitals classified as secondary when the study took place may later become tertiary while those tertiary were seldom downgraded. Besides, we did not contact authors to request on incomplete data, and studies with missing information on selected characteristics were not included in corresponding subgroup analyses, which may introduce biases if the missing did not occur at random. In addition, the potential impacts of some factors (e.g., funding source) on the pooled results were not explored in the present study. Moreover, most of the included studies did not clarify how gestational age was determined, and the heaping of gestational age was not documented in the original studies, so we could not exclude likelihood of some biases of our pooled rates [1]. It is also notably that the heterogeneity across population-based studies, across hospital-based studies, and even across most subgroups of these two types of studies, was very high. Future studies are encouraged to explore other potential sources of heterogeneity beyond those explored in the present study, such as obstetrical factors like maternal age at delivery and parity.

## Conclusions

The pooled analysis of population-based studies with a wide geographic coverage generates a relatively lower preterm delivery rate of around 5% in China. More than three-fourths of studies on China’s preterm delivery rate in the literature are hospital-based. Pooled estimation without discrimination between hospital- and population-based studies will inevitably overestimate the burden of preterm delivery and therefore is not warranted. Large scale population-based studies are needed to obtain more accurate estimates of preterm delivery rate in China.

## Supplementary Information

**Additional file 1.** 

## Data Availability

All data generated or analysed during this study are included in this published article and its supplementary information file.

## Abbreviations

WHO: World Health Organization
PRISMA: Preferred Reporting Items for Systematic Reviews and Meta-Analyses
JBI: Joanna Briggs Institute
CI: Confidence Interval
