# Supplementary material for: Preterm delivery rate in China: a systematic review and meta-analysis
Source: BMC Pregnancy Childbirth. 2022 May 2;22:383. doi: 10.1186/s12884-022-04713-z (PMC9063297; doi:10.1186/s12884-022-04713-z)
Supplement: Supplementary file 1 — Additional file 1. [file 12884_2022_4713_MOESM1_ESM.pdf]

Supplementary appendix

Content

eText1. Full search strategy......2

eText2. List of included studies (n=162)......3

eTable1 PRISMA Checklist......14

eTable2. Characteristics of included studies (n=162)......16

eTable3. Study quality assessment (n=162)......26

eTable4. Stratified analysis within population-based studies (n=37)......36

eTable5. Stratified analysis within hospital-based studies (n=124)......38

eTable6. Province-level pooled rates and nationwide weighted rate......40

eFigure1. Forest plot of all the included studies (n=161)......42

eFigure2. Sensitivity analysis within population-based studies (n=37)......43

eText1. Full search strategy

PubMed

preterm birth[MeSH Terms] AND China[MeSH Terms] AND (incidence[MeSH Terms] OR risk factors[MeSH Terms] OR prevalence[MeSH Terms])

Embase

All field	preterm OR pre-term OR premature OR pre-mature OR prematuritas OR prematurity OR pre-maturity
AND All field	birth OR childbirth OR deliver OR delivery OR labor OR labour
AND Title or Abstract	China OR Chinese
AND All field	prevalence OR incidence OR rate OR risk factors

Web of Science

TS= ((preterm OR pre-term OR premature OR pre-mature OR prematuritas OR prematurity OR pre-maturity) AND (birth OR childbirth OR deliver OR delivery OR labor OR labour) AND ((prevalence OR incidence OR rate) OR risk factors)) AND TI= (China OR Chinese)

China National Knowledge Infrastructure

SU = ('流行' +'发生率'+ '影响因素'+ '出生结局') AND(TI='早产' OR KY='早产')

China Science and Technology Journal Database

M=早产 AND (U=流行 OR U=发生率 OR U=影响因素 OR U=出生结局)

Wanfang Data

题名或关键词:(早产) *题名或关键词:(流行+影响因素+发生率+出生结局)

eText2. List of included studies (n=162)

1. Hu CX, Li YP, Hua SP, et al. [Analysis of the related factors and neonatal outcomes of 470 cases of preterm deliveries]. *Mat Child Heal Care of China* 2013;28(18):2914-16.
2. Zhu T, Liu TJ, Ge X, et al. High prevalence of maternal vitamin D deficiency in preterm births in northeast China, Shenyang. *International journal of clinical and experimental pathology* 2015;8(2):1459-65.
3. Kong DC, Liu HY, Bu YC, et al. [The effect of chronic hepatitis B virus infection(CHB)during pregnancy on the neonatal growth and development]. *Fudan Univ J Med Sci* 2014;41(3):298-303. doi: 10.3969/j.issn.1672-8467.2014.03.003
4. Zhou SL, Liu CX, Cui H. [Changes in the risk factors for preterm delivery between 2001 and 2011]. *J China Med Univ* 2013;42(08):751-54.
5. Reyihanguli MMT, Liu HY, Han WH, et al. [Influence of intrahepafic cholestasis during pregnancy on the incidence of preterm birth]. *Chin J Epidemiol* 2017;38(10):1415-18.
6. Guo YW, Mao SF, Zhu YJ, et al. [The analysis of the occurrence and influence factors of premature]. *Chin J Coal Indust Med* 2017;20(10):1240-46.
7. Yang YX. [Clinical analysis of maternal-fetal outcome and infection caused by premature rupture of membranes] [dissertation]. Shanxi (CN): Shanxi Medical University, 2016.
8. Li YH. [Cause analysis of iatrogenic preterm birth in recent 5 years]. *J Math Med* 2016;29(03):391-93.
9. Lei Q, Niu J, Lv L, et al. Clustering of metabolic risk factors and adverse pregnancy outcomes: a prospective cohort study. *Diabetes/Metabolism Research and Reviews* 2016;32(8):835-42. doi: 10.1002/dmrr.2803
10. Liu WY, Yu ZB, Qiu HY, et al. Association between ambient air pollutants and preterm birth in Ningbo, China: A time-series study. *BMC Pediatrics* 2018;18(1) doi: 10.1186/s12887-018-1282-9
11. Guo Y, Long X, Yao S. [Correlation between the myometrial thickness in the second trimester and preterm delivery in a prospective study]. *Chin J Obstet Gynecol* 2015;50(2):108-11.
12. Lei Q, Niu JM, Duan DM, et al. [Analysis of maternal influencing factors of adverse outcomes in perinatal infants]. *Guangdong Med J* 2014;35(13):2041-45.
13. Zhu H, Yang M-J. Maternal plasma concentrations of macrophage migration inhibitory factor at first trimester as a predictive biomarker of preterm delivery in Chinese women. *Clinica Chimica Acta* 2018;483:286-90. doi: 10.1016/j.cca.2018.04.029
14. Zhang Y, Gu N, Wang Z, et al. Use of the 10-Group Classification System to analyze how the population control policy change in China has affected cesarean delivery. *International Journal of Gynecology and Obstetrics* 2017;138(2):158-63. doi: 10.1002/ijgo.12210
15. Zhang JJ. [Study on the influencing factors of preterm birth and low birth weight] [dissertation]. Gansu (CN): Lanzhou University, 2013.
16. Huang HF, Zhong WH, Zhou QN, et al. [Analysis of the occurrence of premature infants hospitalized in the hospital in

2014 and 2016]. *Chin J Rural Med Pharm* 2018;25(13):49.

17. Xiong JJ. [Effect of hepatitis B virus infection with abnormal liver function on preterm delivery in pregnant women]. *Mod Diagn Treat* 2017;28(11):2068-69.
18. Qin J, Sheng X, Wu D, et al. Adverse obstetric outcomes associated with in vitro fertilization in singleton pregnancies. *Reproductive sciences (Thousand Oaks, Calif)* 2017;24(4):595-608. doi: 10.1177/1933719116667229
19. Zhao Q-M, Liu F, Wu L, et al. Prevalence of congenital heart disease at live birth in China. *Journal of Pediatrics* 2019;204:53-58. doi: 10.1016/j.jpeds.2018.08.040
20. Zhu L, Zhang R, Zhang S, et al. [Chinese neonatal birth weight curve for different gestational age]. *Chin J Pediatr* 2015;53(2):97-103.
21. Wang RH, Zhang L, Wang LN, et al. [Adverse pregnancy outcomes and ambient air pollution in two urban areas of Gansu province]. *Chin J Public Health* 2018;34(01):75-78.
22. Yan Y, Liu Z, Liu D. Heterogeneity of glycometabolism in patients with gestational diabetes mellitus: Retrospective study of 1,683 pregnant women. *Journal of Diabetes Investigation* 2017;8(4):554-59. doi: 10.1111/jdi.12595
23. Fan LC. [Study on the association between air pollutants exposure and adverse pregnancy outcomes in Hainan province] [dissertation]. Hubei (CN): Huazhong University of Science and Technology, 2013.
24. Ji DY, Wang J. [Relationship between birth order of live neonates and premature and low birth weight: A cross-sectional study due to the two-child policy]. *Anhui Med J* 2015;36(6):704-07.
25. Liu L. [Periodontal status of 702 pregnant women and relationship between maternal periodontitis with preterm birth and low birth weight] [dissertation]. Anhui (CN): Anhui Medical University, 2012.
26. Xu H, Dai Q, Xu Y, et al. Time trends and risk factor associated with premature birth and infants deaths due to prematurity in Hubei Province, China from 2001 to 2012. *BMC Pregnancy and Childbirth* 2015;15(1) doi: 10.1186/s12884-015-0767-x
27. Hou XJ, Wu LP, Zhang RR, et al. [Relationship between multi-vitamins supplementation during the second and third trimester and risk of preterm delivery]. *Chin J Woman Child Heal Res* 2018;29(04):481-84.
28. Peng TT. [Classification tree model for risk factors of preterm birth and interaction analysis] [dissertation]. Shanxi (CN): Shanxi Medical University, 2015.
29. Zhao XL, Liao Y, Li LM, et al. [Retrospective cohort study on rapid body mass index gain in infants from birth to 1 year old]. *Chin J Child Heal Care* 2018;26(03):258-61.
30. Zhang XB, Li XY, Huang Y, et al. [Clinical analysis of 1100 cases in premature labor]. *Chin J Birth Heal & Hered* 2015;23(07):52-53+120.
31. Yu YH, Huang QT, Xu JZ, et al. [Investigation of preterm birth and preterm infants in Guangzhou regional district]. *China Mod Med* 2012;19(22):13-15+18.
32. Liu X, Lv L, Zhang H, et al. Folic acid supplementation, dietary folate intake and risk of preterm birth in China. *European Journal of Nutrition* 2016;55(4):1411-22. doi: 10.1007/s00394-015-0959-1
33. Xu F, Kong X, Duan S, et al. Respiratory distress syndrome in premature infants: A retrospective cohort study from 14

hospitals in China. *International Journal of Clinical and Experimental Medicine* 2019;12(6):7497-506.

34. Liu XY, Xu LF, Jin ZB, et al. [Incidence of late preterm infants and clinical problem analysis during perinatal period]. *China Mod Med* 2014;21(26):22-23+26.

35. Zhu Y. [A prospective multicenter epidemiology investigation of premature newborn] [dissertation]. Shanghai (CN): Fudan University, 2012.

36. Zhang XS, Zhao GL, Yang HX, et al. [Analysis of incidence and risk factors of preterm birth in 15 urban hospitals in China]. *Chin J Perinat Med* 2016;19(6):456-61.

37. Zhou M. [Influence factors of body mass index before pregnancy and the relationship with gestational weight gain,maternal and infants complications]. *Chin J Perinat Med* 2015;18(5):352-58.

38. Multicenter Collaborative Team for the Respiratory Distress Syndrome. [Respiratory distress syndrome in inborn preterm neonates: A retrospective investigation in 13 hospitals in China]. *Chin J Perinat Med* 2016;19(10):743-54.

39. Han Y, Jiang P, Dong T, et al. Maternal air pollution exposure and preterm birth in Wuxi, China: Effect modification by maternal age. *Ecotoxicology and Environmental Safety* 2018;157:457-62. doi: 10.1016/j.ecoenv.2018.04.002

40. Chen C, Zhang JW, Xia HW, et al. Preterm birth in China between 2015 and 2016. *Am J Public Health* 2019;109(11):1597-604.

41. Sun P-F, Ding G-C, Zhang M-Y, et al. Prevalence of congenital heart disease among infants from 2012 to 2014 in Langfang, China. *Chinese Medical Journal* 2017;130(9):1069-73. doi: 10.4103/0366-6999.204923

42. Jiang F, Gao JS, Zhong YF, et al. [Preterm births in peking union medical college hospital in the past 25 years]. *Acta Academiae Medicinae Sinicae* 2016;38(5):528-33. doi: 10.3881/j.issn.1000-503X.2016.05.006

43. Zhou X, Yin Y, Zhang GY, et al. [Clinical analysis of 1 466 cases of preterm birth from 2008 to 2012]. *J Int Obstet Gynecol* 2014;41(04):434-37.

44. Liu XJ, Lu LJ, Qiu H, et al. [Epidemiological analysis of complications in preterm infants]. *Chin J Prim Med Pharm* 2019;26(3):315-18.

45. Li PC, Liao ZJ, Wang C, et al. [Maternal characteristics and birth outcomes under different family planning policy]. *Chin J Reprod Heal* 2017;28(6):501-05.

46. Yu H, Jiang X, Bao W, et al. Association of intimate partner violence during pregnancy, prenatal depression, and adverse birth outcomes in Wuhan, China. *Bmc Pregnancy and Childbirth* 2018;18 doi: 10.1186/s12884-018-2113-6

47. Tian Q, Wu BQ, Liu XH, et al. [Epidemiologic study of neonatal respiratory distress syndrome in Shenzhen area]. *Chin J Appl Clin Pediatr* 2013;28(14):1083-85.

48. Zhong SL, Deng YQ. [Clinical analysis on the changes of high risk factors related to premature delivery in the hospital from 2005 to 2014]. *J Qiqihar Univ Med* 2016;37(02):235-37.

49. Ke L, Lin W, Liu Y, et al. Association of induced abortion with preterm birth risk in first-time mothers. *Scientific reports* 2018;8(1):5353. doi: 10.1038/s41598-018-23695-7

50. Liu WL, Yao L, Cao SH, et al. [Influencing factors of preterm birth under regional differences]. *Contemp Med*

2016;22(1):3-5.

51. Shen CR. [Analysis of the influence of hypertensive diseases in pregnancy on birth outcome and related factors]. *Chin J Hypertens* 2015;23(2):169-70.

52. Barros FC, Papageorghiou AT, Victora CG, et al. The distribution of clinical phenotypes of preterm birth syndrome implications for prevention. *JAMA Pediatrics* 2015;169(3):220-29. doi: 10.1001/jamapediatrics.2014.3040

53. Zhao QQ. [Clinical analysis of 930 cases of preterm birth] [dissertation]. Liaoning (CN): Dalian Medical University, 2016.

54. Zhou J, Su L, Liu M, et al. Associations between 25-hydroxyvitamin D levels and pregnancy outcomes: A prospective observational study in southern China. *European Journal of Clinical Nutrition* 2014;68(8):925-30. doi: 10.1038/ejcn.2014.99

55. Dong RJ. [The differences occurred of premature delivery in Korean and Han nationalities and relationship between IL-6, TNF-α in umbilical cord blood of neonates with PPROM and chorioamnionitis] [dissertation]. Jilin (CN): Yanbian University, 2017.

56. Feng FF, Lei T, Wu Q. [Risk factors of preterm birth and its influence on the fetus]. *Med Inform* 2013;26(8):138-38.

57. Zhuang X, Cui AM, Wang Q, et al. Liver dysfunction during pregnancy and its association of with preterm birth in China: A prospective cohort study. *EBioMedicine* 2017;26:152-56. doi: 10.1016/j.ebiom.2017.11.014

58. Liu Y, Li G, Zhang W. Effect of fetal gender on pregnancy outcomes in Northern China. *Journal of Maternal-Fetal & Neonatal Medicine* 2017;30(7):858-63. doi: 10.1080/14767058.2016.1189527

59. Sun JH. [Prospective study of obstetrics and gynecology on the relationship between artificial abortion and premature delivery]. *Guide China Med* 2013;11(26):12-13.

60. Yan JM, Huang H, Li QQ, et al. [A single-center study on the incidence and mortality of preterm infants from 2006 to 2016]. *Chin J Contemp Pediatr* 2018;20(5):368-72.

61. Zeng LX. [Investigation and analysis on the prevalence and outcome of premature infants after the implementation of the two-child policy]. *J Xiangnan Univ (Med Sci)* 2017;19(4):53-55.

62. Tan J, Huang S, He G, et al. Maternal hepatitis B surface antigen carrier status and its impact on neonatal outcomes: A cohort study of 21 947 singleton newborns in China. *Journal of Maternal-Fetal & Neonatal Medicine* 2017;30(18):2219-24. doi: 10.1080/14767058.2016.1243098

63. Guo TJ, Zhang Y, Wang YY, et al. [Influence of extreme ambient temperature exposure on preterm birth]. *Chin J Woman Child Heal Res* 2017;28(12):1521-24.

64. Chen P. [Statistical analysis of the correlation between pre-pregnancy body mass index and weight gain during pregnancy and preterm birth]. *Chin J Hos Stat* 2015;22(02):116-17.

65. Liu L, Hong Z, Zhang L. Associations of prepregnancy body mass index and gestational weight gain with pregnancy outcomes in nulliparous women delivering single live babies. *Scientific reports* 2015;5:12863. doi: 10.1038/srep12863

66. Huang X, Tan H, Cai M, et al. Gestational weight gain in Chinese women - results from a retrospective cohort in Changsha, China. *Bmc Pregnancy and Childbirth* 2018;18 doi: 10.1186/s12884-018-1833-y

67. Tang W, Mu Y, Li X, et al. Low birthweight in China: Evidence from 441 health facilities between 2012 and 2014. *Journal of Maternal-Fetal and Neonatal Medicine* 2017;30(16):1997-2002. doi: 10.1080/14767058.2016.1236081

68. Xu R, Wang J, Liu JN, et al. [The trend and risk factors of preterm birth and low birth weight in Suzhou]. *Mat Child Heal Care of China* 2016;31(22):4793-96.

69. Lu RX. [Investigation of preterm status quo and prognosis of preterm of Fuyang City]. *China Mod Doctor* 2012;50(33):21-23.

70. Wang XJ, Wen XL, Xie WH. [An epidemiological survey of premature infants in Guangzhou Women and Children Medical Center]. *Shenzhen J Integ Trad Chin West Med* 2018;28(11):16-17.

71. Wang S, Wang T, Zhang W, et al. Cohort study on maternal cytomegalovirus seroprevalence and prevalence and clinical manifestations of congenital infection in China. *Medicine* 2017;96(5) doi: 10.1097/md.0000000000006007

72. Yi L, Zhang B, Han Q, et al. [Study on the neonatal conditions in four cities of China in 2012]. *Journal of Clinical Pediatrics* 2013;31(10):917-20.

73. Du HJ, Zhao ZY, Ci LJ, et al. [A survey of newborns in the hospital in 2013]. *Chin J of Clinical Rational Drug Use* 2016;9(02):138-40.

74. Guo T, Wang Y, Zhang H, et al. The association between ambient temperature and the risk of preterm birth in China. *Science of the Total Environment* 2018;613:439-46. doi: 10.1016/j.scitotenv.2017.09.104

75. Ding J, Zhang JX. [Analysis on the influencing factors of premature delivery in Beijing Hui Min Hospital]. *Chin Baby* 2016(4):7-8.

76. Luo XL, Zhang WY. Obstetrical disease spectrum in China: An epidemiological study of 111,767 cases in 2011. *Chinese Medical Journal* 2015;128(9):1137-46. doi: 10.4103/0366-6999.156076

77. Liu YW. [Study on the effects of blood pressure changes before and after pregnancy on pregnancy outcome] [dissertation]. Hebei (CN): North China University of Science and Technology, 2018.

78. Kang HX, Ma CH, Wei Y, et al. [Analysis of the relationship between the risk of preterm delivery in singleton pregnancy and in vitro fertilization and embryo transfer]. *Chin J Prac Gynecol Obstet* 2017;33(06):609-12.

79. Yuan X, Hu H, Zhang M, et al. Iron deficiency in late pregnancy and its associations with birth outcomes in Chinese pregnant women: A retrospective cohort study. *Nutrition & Metabolism* 2019;16 doi: 10.1186/s12986-019-0360-9

80. Xue Q, Shen F, Gao Y, et al. An analysis of the medical indications for preterm birth in an obstetrics and gynaecology teaching hospital in Shanghai, China. *Midwifery* 2016;35:17-21. doi: 10.1016/j.midw.2016.01.013

81. Liu JL. [Effect of early perinatal care on preterm birth prevention and preterm birth prevalence reduction]. *Chin Fore Women Heal* 2013(02X):103-04.

82. Xu Q, Yang Y, Wang L, et al. Preconception alanine aminotransferase concentrations and risk of preterm birth in rural Chinese women aged 20-49 years: A population-based cohort study. *Lancet* 2018;392:13-13.

83. Ren DF. [Epidemiology investigation of relevant factors of preterm labour] [dissertation]. Jiangsu (CN): Southeast University, 2013.

84. Shan X, Chen F, Wang W, et al. Secular trends of low birthweight and macrosomia and related maternal factors in Beijing, China: A longitudinal trend analysis. *BMC Pregnancy and Childbirth* 2014;14(1) doi: 10.1186/1471-2393-14-105

85. Yu HT, Jin WZ, Qian NS, et al. [Evaluating newborn's adverse birth outcomes attributable to maternal secondhand smoke exposure in Shanghai]. *J Environ Occup Med* 2016;33(10):954-58.

86. Yan H, Gao S, Li N, et al. Impact of cervical length on preterm birth in northern China: A prospective cohort study. *Journal of Maternal-Fetal and Neonatal Medicine* 2019 doi: 10.1080/14767058.2019.1570116

87. Wang CS, Wei YM, Yang HX. [Analysis of the effects of gestational diabetes mellitus based on abnormal blood glucose on pregnancy outcomes]. *Chin J Obstet Gynecol* 2013;48(12):899-902.

88. Li SS. [A study on the relationship between anxiety,stress and its relationship with preterm delivery] [dissertation]. Guangxi (CN): Guangxi Medical University, 2017.

89. Xin QQ, Chen BW, Yin DL, et al. Prevalence of anemia and its risk factors among children under 36 months old in China. *Journal of Tropical Pediatrics* 2017;63(1):36-42. doi: 10.1093/tropej/fmw049

90. Guan T, Xue T, Gao S, et al. Acute and chronic effects of ambient fine particulate matter on preterm births in Beijing, China: A time-series model. *Science of the Total Environment* 2019;650:1671-77. doi: 10.1016/j.scitotenv.2018.09.279

91. Guo Y, Liu Y, He J-R, et al. Changes in birth weight between 2002 and 2012 in Guangzhou, China. *PloS one* 2014;9(12) doi: 10.1371/journal.pone.0115703

92. Ye L, Ji Y, Lv W, et al. Associations between maternal exposure to air pollution and birth outcomes: A retrospective cohort study in Taizhou, China. *Environmental science and pollution research international* 2018;25(22):21927-36. doi: 10.1007/s11356-018-1944-z

93. Han S, Yang X, Zhou Y, et al. Deafness gene mutations in newborns in Beijing. *Acta oto-laryngologica* 2016;136(5):475-9. doi: 10.3109/00016489.2015.1128121

94. Li C, Liang Z, Bloom MS, et al. Temporal trends of preterm birth in Shenzhen, China: A retrospective study. *Reproductive Health* 2018;15(1) doi: 10.1186/s12978-018-0477-8

95. Yang M, Wang Y, Chen Y, et al. Impact of maternal HIV infection on pregnancy outcomes in southwestern China - a hospital registry based study. *Epidemiology and infection* 2019;147:e124. doi: 10.1017/S0950268818003345

96. Zhang DD, Tan DX, Wang B, et al. [Association between gestational weight gain and preterm birth： A retrospective epidemiological analysis in Wuhan]. *Chin J Epidemiol* 2016;37(7):1012-16.

97. Yu R, Wang FY, Cui ZQ, et al. [Comparative analysis of correlative factors in different types premature delivery]. *Chin J Diffic and Compl Cas* 2015;14(5):501-03.

98. Zhao JQ, Yang N, Gong LF, et al. [Analysis on newborn status of preterm infants in Beijing from 2011 to 2016]. *Chin J Child Heal Care* 2018;26(11):1254-56.

99. Su WJ, Chen YL, Huang PY, et al. Effects of prepregnancy body mass index, weight gain, and gestational diabetes mellitus on pregnancy outcomes: A population-based study in Xiamen, China, 2011-2018. *Annals of nutrition & metabolism* 2019;75(1):31-38. doi: 10.1159/000501710

100. Zheng F. [Risk factor analysis of premature delivery in pregnant women and its influence on neonates]. *Heal Mus Read Mag* 2013(10):500-01.

101. Xia M, Yang P, Cui W, et al. Clinical analysis of 122 Chinese cases of twin pregnancy complicated with preterm birth and outcomes of preterm infants. *International Journal of Clinical and Experimental Medicine* 2017;10(7):10857-65.

102. Xu J, Wang J, Xuan S, et al. The effects of childbirth age on maternal and infant outcomes in pregnant women. *Iranian journal of public health* 2018;47(6):788-93.

103. Wang C, Wang XY, Yang HX. [Effect of maternal age on pregnancy outcomes in Beijing]. *Chin J Obstet Gynecol* 2017;52(8):514-20.

104. Li X, Liu Y, Liu F, et al. Analysis of short-term and sub-chronic effects of ambient air pollution on preterm birth in central China. *Environmental Science and Pollution Research* 2018;25(19):19028-39. doi: 10.1007/s11356-018-2061-8

105. Wei LJ, Liu S, Huang DP, et al. [Dose-response relationship between maternal hemoglobin concentration and preterm birth, in pregnant women]. *Chin J Epidemiol* 2019;40(4):471-74.

106. Ye J. [Hypertension disorder in pregnancy: Prediction and risk for adverse pregnancy outcomes] [dissertation]. Guangxi (CN): Guangxi Medical University, 2017.

107. Xu HR. [Clinical study of early perinatal health care to prevent premature delivery and reduce the prevalence of premature delivery]. *Chin J Heal Care Nutri* 2013;1(1):119-19.

108. Lin XH, Wu DD, Li C, et al. Maternal high triglyceride levels during early pregnancy and risk of preterm delivery: A retrospective cohort study. *Journal of Clinical Endocrinology and Metabolism* 2019;104(4):1249-58. doi: 10.1210/jc.2018-01372

109. Hu R, Chen Y, Zhang Y, et al. Association between vomiting in the first trimester and preterm birth: A retrospective birth cohort study in Wuhan, China. *Bmj Open* 2017;7(9) doi: 10.1136/bmjopen-2017-017309

110. Sun B, Wang D. [Impact of perinatal risks and social, cultural and technological factors on outcome of preterm neonates]. Proceedings of the 5th Shanghai Neonatal Forum. 2015 Apr 9-11; Shanghai, China:2015. p. 57-59.

111. Guo P, Miao H, Chen Y, et al. Maternal exposure to gaseous ambient air pollutants increases the risk of preterm birth in the Pearl River Delta, China 2014-2017. *Science of the Total Environment* 2019;671:959-70. doi: 10.1016/j.scitotenv.2019.03.375

112. Liu T, Qiu XQ, Zeng XY, et al. [Influencing factors of premature delivery of pregnant women in Guangxi region and the relationship between premature birth and basic characteristics of perinatal infant]. *Mat Child Heal Care of China* 2019;34(11):2596-601.

113. He JR, Ramakrishnan R, Lai YM, et al. Predictions of preterm birth from early pregnancy characteristics: Born in Guangzhou cohort study. *Journal of clinical medicine* 2018;7(8):185. doi: 10.3390/jcm7080185

114. Yang W, Han F, Gao X, et al. Relationship between gestational weight gain and pregnancy complications or delivery outcome. *Scientific reports* 2017;7(1):12531. doi: 10.1038/s41598-017-12921-3

115. Liu S, Yan Y, Gao X, et al. Risk factors for postpartum depression among Chinese women: Path model analysis. *BMC*

Pregnancy and Childbirth 2017;17(1) doi: 10.1186/s12884-017-1320-x

116. Xiao Y, Sun H, Li C, et al. Effect of iodine nutrition on pregnancy outcomes in an iodine-sufficient area in China. *Biological Trace Element Research* 2018;182(2):231-37. doi: 10.1007/s12011-017-1101-4

117. li GJ. [Analysis of risk factors of 202 premature births]. *Guide China Med* 2013;11(31):162-64.

118. Dai XW, Li XX. [Study on the relationship between depression during pregnancy and premature delivery]. *Mat Child Heal Care of China* 2019;34(4):792-96.

119. Wang C, Lin L, Su R, et al. Hemoglobin levels during the first trimester of pregnancy are associated with the risk of gestational diabetes mellitus, pre-eclampsia and preterm birth in Chinese women: A retrospective study. *Bmc Pregnancy and Childbirth* 2018;18 doi: 10.1186/s12884-018-1800-7

120. Tang JB. [Analysis of epidemiology and related risk factors of low birth weight infants] [dissertation]. Zhejiang (CN): Ningbo University, 2018.

121. Li R, Zhang J, Zhou R, et al. Sleep disturbances during pregnancy are associated with cesarean delivery and preterm birth. *The journal of maternal-fetal & neonatal medicine: the official journal of the European Association of Perinatal Medicine, the Federation of Asia and Oceania Perinatal Societies, the International Society of Perinatal Obstetr* 2017;30(6):733-38. doi: 10.1080/14767058.2016.1183637

122. Wang YC. [An epidemiological study on the birth and growth of preterm infants in the Wenzhou area in 2014] [dissertation]. Zhejiang (CN): Wenzhou Medical University, 2018.

123. Pan L, Leng J, Liu G, et al. Pregnancy outcomes of Chinese women with gestational diabetes mellitus defined by the IADPSG's but not by the 1999 WHO's criteria. *Clinical Endocrinology* 2015;83(5):684-93. doi: 10.1111/cen.12801

124. Hu J, Li Y, Zhang B, et al. Impact of the 2017 ACC/AHA Guideline for high blood pressure on evaluating gestational hypertension-associated risks for newborns and mothers. *Circulation research* 2019;125(2):184-94. doi: 10.1161/circresaha.119.314682

125. Xiao Q, Chen H, Strickland MJ, et al. Associations between birth outcomes and maternal PM2.5 exposure in Shanghai: A comparison of three exposure assessment approaches. *Environment international* 2018;117:226-36. doi: 10.1016/j.envint.2018.04.050

126. He JF, Xiong HY, Li L. [Clinical features of premature birth: A report of 599 cases from a hospital]. *J Third Mil Med Univ* 2014;36(9):966-70.

127. Xue XP, Su B. [Analysis on the influencing factors for preterm birth, Taiyuan, 2009-2010]. *Prev Med Trib* 2015;21(6):408-11.

128. Xu X, Tan HZ, Zhou SJ, et al. [Study on the application of Back-Propagation Artificial Neural Network used the model in predicting preterm birth]. *Chin J Epidemiol* 2014;35(9):1028-31.

129. Yang Q, Zhou ZX, Wang F, et al. [Discussion on the related factors and preventive measures of premature delivery]. *Mat Child Heal Care of China* 2012;27(15):2305-06+72.

130. Long XZ. [Fetal fibronectin quantitative clinical observations and research]. *M M I* 2016;25(03):600-03.

131. Guo YW, Tan HZ, Zhou SJ, et al. [A population based study on incidence and determinants of preterm birth in Liuyang Hunan]. *J Cent South Univ (Med Sci)* 2013;38(4):413-18.

132. Zhang CH, Liu XY, Zhan YW, et al. Effects of prepregnancy body mass index and gestational weight gain on pregnancy outcomes. *Asia-Pacific journal of public health* 2015;27(6):620-30. doi: 10.1177/1010539515589810

133. Zhu B, Huang K, Bao W, et al. Dose-response relationship between maternal blood pressure in pregnancy and risk of adverse birth outcomes: Ma'anshan birth cohort study. *Pregnancy hypertension* 2019;15:16-22. doi: 10.1016/j.preghy.2018.09.004

134. Ding HY, Xiamusiye MYDL, Li MC, et al. [Analysis of trends of birth weight， and incidence of adverse birth outcomes and relevant factors from 2002 to 2015]. *Chin J Dis Control Prev* 2018;22(4):380-85.

135. Wang F, Zhang HL. [The prevalence and risk factors of preterm birth in Ezhou city from 2010 to 2014]. *Chin J Social Med* 2016;33(3):252-54,55. doi: 10.3969/j.issn.1673-5625.2016.03.017

136. Ma HH, Jin CY, Wang HJ, et al. [Prevalence and risk factors of preterm birth among singletons from2013 to 2017 in Tongzhou district of Beijing]. *Chin J Child Heal Care* 2019;27(06):592-96.

137. Zhou M, Zhang T, Huang AQ, et al. [Relationship between normative antenatal care and preterm birth]. *Chin J Woman Child Heal Res* 2015;26(3):422-25.

138. Tian JR, Tang WJ, Su C, et al. [Study of risk factors of preterm delivery in Qinzhou, Guangxi]. *Applied Prev Med* 2018;24(03):173-77.

139. Zhao YJ. [The changing trends of birth outcomes in Taicang, Jiangsu from 2009 to 2015 and their risk factor analysis] [dissertation]. Beijing (CN): Chinese Center for Disease Control and Prevention, 2017.

140. Wang H, Yue H, Sun B, et al. Birth population survey in Huai'an in 2015: Perinatal-neonatal mortality and preterm birth rate in emerging regions in China. *Journal of Maternal-Fetal and Neonatal Medicine* 2018 doi: 10.1080/14767058.2018.1506439

141. Hu CY, Li FL, Jiang W, et al. Pre-pregnancy health status and risk of preterm birth: A large, Chinese, rural, population-based study. *Medical Science Monitor* 2018;24:4718-27. doi: 10.12659/MSM.908548

142. Zheng W, Huang W, Zhang L, et al. Changes in Serum Lipid Levels During Pregnancy and Association With Neonatal Outcomes: A Large Cohort Study. *Reproductive Sciences* 2018;25(9):1406-12. doi: 10.1177/1933719117746785

143. Huang A, Wu K, Zhao W, et al. Attendance at prenatal care and adverse birth outcomes in China: A followup study based on Maternal and Newborn's Health Monitoring System. *Midwifery* 2018;57:26-31. doi: 10.1016/j.midw.2017.10.018

144. Fei L. [The prevalence of preterm birth and its influencing factors in Gongyi]. *Mat Child Heal Care of China* 2014;29(14):2152-53.

145. Fang HQ, Xu H. [Occurrence of low birth weight infants and its influencing factors in Yandu district of Yancheng in 2015]. *Chin Nurs Res* 2017;31(1):359-61.

146. Yang J, Liu Y, Liu H, et al. Associations of maternal iodine status and thyroid function with adverse pregnancy outcomes in Henan Province of China. *Journal of Trace Elements in Medicine and Biology* 2018;47:104-10. doi:

10.1016/j.jtemb.2018.01.013

147. Wen T, Lv Y. Inadequate gestational weight gain and adverse pregnancy outcomes among normal weight women in China. *International Journal of Clinical and Experimental Medicine* 2015;8(2):2881-86.

148. Jia XM. [Maternal psychosocial stress in the first trimester of pregnancy and premature birth: A follow up study] [dissertation]. Anhui (CN): Anhui Medical University, 2015.

149. Chen LM, Du WJ, Dai J, et al. Effects of subclinical hypothyroidism on maternal and perinatal outcomes during pregnancy: A single-center cohort study of a Chinese population. *PloS one* 2014;9(10):e109364. doi: 10.1371/journal.pone.0109364

150. Fan XJ, Gao JM, Kang YJ, et al. Socioeconomic and environmental determinants to preterm birth in Tibetan women: An analysis based on the hierarchically conceptual frame. *Chinese medical journal* 2017;130(19):2307-15. doi: 10.4103/0366-6999.215341

151. Fang J, Liu H, Zhao H, et al. Association of in utero hexachlorocyclohexane exposure with gestational age. *Ecotoxicology and environmental safety* 2019;174:263-69. doi: 10.1016/j.ecoenv.2019.02.089

152. Li Y, Chen X, Chen S, et al. [A cohort study on the impacts of pre-pregnancy maternal body mass index, gestational weight gain on neonate birth status and perinatal outcomes in Fujian province]. *Chin J Epidemiol* 2014;35(6):635-40.

153. Li MM, Li C, Zhang BY, et al. [Relationship between preterm birth of infant and medication during pregnancy in women of childbearing age in Shaanxi]. *Chin J Epidemiol* 2019;40(5):554-58.

154. Hu Y, Li L, Huang WZ, et al. [Retrospective study of risk factors for premature birth in our hospital in recent 10 years]. *Hainan Med J* 2016;27(20):3356-58.

155. Jin WY, Lin SL, Hou RL, et al. Associations between maternal lipid profile and pregnancy complications and perinatal outcomes: A population-based study from China. *BMC pregnancy and childbirth* 2016;16:60. doi: 10.1186/s12884-016-0852-9

156. Zhao X. [A study on the correlation between the different levels of weight gain in pregnancy and the effect of pregnancy outcome] [dissertation]. Shandong (CN): Qingdao University, 2018.

157. Guo T, Wang Y, Zhang Y, et al. Association between PM2.5 exposure and the risk of preterm birth in Henan, China: A retrospective cohort study. *The Lancet* 2017;390(SPEC.ISS 1):24.

158. Guo YW, Mao SF. [The retrospective of preterm birth outcomes and macrosomia pregnancy in hospital of Chengde in 2010]. *Hebei Med* 2016;22(10):1752-54.

159. Wang J. [Analysis of relationship between premature rupture of membranes and maternal and fetal complications]. *J China Prescri Drug* 2014;12(02):60-61.

160. Jiang Y. [Analysis of relevant risk factors for premature delivery of 5000 pregnant women in Guzhen area of Zhongshan city in Guangdong province]. *China Mod Med* 2016;23(01):137-40.

161. Wang M, Zhang FX, Li M. [Study on the relationship between prevention and treatment of gingivitis during pregnancy and the prevention of premature delivery and low birth weight infants]. *Ningxia Med J* 2013;35(12):1238-39.

162. Wang FX, Ding YH, Xu L. [Analysis of eugenics health examination results of pre pregnancy in Zhejiang province]. *Prev Med* 2019;31(07):742-43+47.

PRISMA 2020 Checklist

Section and Topic	Item #	Checklist item	Location where item is reported
**TITLE**			
Title	1	Identify the report as a systematic review.	P1
**ABSTRACT**			
Abstract	2	See the PRISMA 2020 for Abstracts checklist.	P1
**INTRODUCTION**			
Rationale	3	Describe the rationale for the review in the context of existing knowledge.	P1-P2
Objectives	4	Provide an explicit statement of the objective(s) or question(s) the review addresses.	P2
**METHODS**			
Eligibility criteria	5	Specify the inclusion and exclusion criteria for the review and how studies were grouped for the syntheses.	P2
Information sources	6	Specify all databases, registers, websites, organisations, reference lists and other sources searched or consulted to identify studies. Specify the date when each source was last searched or consulted.	P2
Search strategy	7	Present the full search strategies for all databases, registers and websites, including any filters and limits used.	eText1
Selection process	8	Specify the methods used to decide whether a study met the inclusion criteria of the review, including how many reviewers screened each record and each report retrieved, whether they worked independently, and if applicable, details of automation tools used in the process.	P2
Data collection process	9	Specify the methods used to collect data from reports, including how many reviewers collected data from each report, whether they worked independently, any processes for obtaining or confirming data from study investigators, and if applicable, details of automation tools used in the process.	P2-P3
Data items	10a	List and define all outcomes for which data were sought. Specify whether all results that were compatible with each outcome domain in each study were sought (e.g. for all measures, time points, analyses), and if not, the methods used to decide which results to collect.	P2-P3
	10b	List and define all other variables for which data were sought (e.g. participant and intervention characteristics, funding sources). Describe any assumptions made about any missing or unclear information.	P2-P3
Study risk of bias assessment	11	Specify the methods used to assess risk of bias in the included studies, including details of the tool(s) used, how many reviewers assessed each study and whether they worked independently, and if applicable, details of automation tools used in the process.	P3
Effect measures	12	Specify for each outcome the effect measure(s) (e.g. risk ratio, mean difference) used in the synthesis or presentation of results.	P3
Synthesis methods	13a	Describe the processes used to decide which studies were eligible for each synthesis (e.g. tabulating the study intervention characteristics and comparing against the planned groups for each synthesis (item #5)).	P3
	13b	Describe any methods required to prepare the data for presentation or synthesis, such as handling of missing summary statistics, or data conversions.	P3
	13c	Describe any methods used to tabulate or visually display results of individual studies and syntheses.	P3
	13d	Describe any methods used to synthesize results and provide a rationale for the choice(s). If meta-analysis was performed, describe the model(s), method(s) to identify the presence and extent of statistical heterogeneity, and software package(s) used.	P3
	13e	Describe any methods used to explore possible causes of heterogeneity among study results (e.g. subgroup analysis, meta-regression).	P3
	13f	Describe any sensitivity analyses conducted to assess robustness of the synthesized results.	P3
Reporting bias assessment	14	Describe any methods used to assess risk of bias due to missing results in a synthesis (arising from reporting biases).	P3

PRISMA 2020 Checklist

Section and Topic	Item #	Checklist item	Location where item is reported
Certainty assessment	15	Describe any methods used to assess certainty (or confidence) in the body of evidence for an outcome.	P3
**RESULTS**			
Study selection	16a	Describe the results of the search and selection process, from the number of records identified in the search to the number of studies included in the review, ideally using a flow diagram.	P3, Fig1
	16b	Cite studies that might appear to meet the inclusion criteria, but which were excluded, and explain why they were excluded.	P3
Study characteristics	17	Cite each included study and present its characteristics.	eText2, eTable2
Risk of bias in studies	18	Present assessments of risk of bias for each included study.	P4, eTable3
Results of individual studies	19	For all outcomes, present, for each study: (a) summary statistics for each group (where appropriate) and (b) an effect estimate and its precision (e.g. confidence/credible interval), ideally using structured tables or plots.	eFig1, Fig3
Results of syntheses	20a	For each synthesis, briefly summarise the characteristics and risk of bias among contributing studies.	P3-P4
	20b	Present results of all statistical syntheses conducted. If meta-analysis was done, present for each the summary estimate and its precision (e.g. confidence/credible interval) and measures of statistical heterogeneity. If comparing groups, describe the direction of the effect.	P3-P5
	20c	Present results of all investigations of possible causes of heterogeneity among study results.	P3-P5
	20d	Present results of all sensitivity analyses conducted to assess the robustness of the synthesized results.	P4
Reporting biases	21	Present assessments of risk of bias due to missing results (arising from reporting biases) for each synthesis assessed.	P4
Certainty of evidence	22	Present assessments of certainty (or confidence) in the body of evidence for each outcome assessed.	P3-P5
**DISCUSSION**			
Discussion	23a	Provide a general interpretation of the results in the context of other evidence.	P5-P6
	23b	Discuss any limitations of the evidence included in the review.	P7-P8
	23c	Discuss any limitations of the review processes used.	P7-P8
	23d	Discuss implications of the results for practice, policy, and future research.	P7
**OTHER INFORMATION**			
Registration and protocol	24a	Provide registration information for the review, including register name and registration number, or state that the review was not registered.	P2
	24b	Indicate where the review protocol can be accessed, or state that a protocol was not prepared.	P2
	24c	Describe and explain any amendments to information provided at registration or in the protocol.	P2
Support	25	Describe sources of financial or non-financial support for the review, and the role of the funders or sponsors in the review.	P8
Competing interests	26	Declare any competing interests of review authors.	P8
Availability of data, code and other materials	27	Report which of the following are publicly available and where they can be found: template data collection forms; data extracted from included studies; data used for all analyses; analytic code; any other materials used in the review.	P8

From: Page MJ, McKenzie JE, Bossuyt PM, et al. The PRISMA 2020 statement: an updated guideline for reporting systematic reviews. *BMJ* 2021;372:n71. doi:10.1136/bmj.n71

For more information, visit: www.prisma-statement.org.

eTable2. Characteristics of included studies (n=162)

No.	Publication year	First author	Language	Study year	Study setting(s)	Region[a]	Data source[b]	HS level	Singleton only	Lower boundary	Upper boundary	Live birth only	Unit of analysis[c]	Total participants	Number of cases	Rate (%)
1	2013	Hu Chunxia	Chinese	2011	Hainan	E	HS	3	No	28	37	No	W	1619	470	29.0
2	2015	Zhu Tong	English	2012	Liaoning	NE	HS	3	Yes	27 or less	37	Yes	N	821	180	21.9
3	2014	Kong Dechuan	Chinese	2012	Anhui	C	HS	3	Yes	27 or less	37	Yes	W	1028	219	21.3
4	2013	Zhou Shenglan	Chinese	2011	Liaoning	NE	HS	3	No	28	37	U[d]	W	6063	1123	18.5
5	2017	Reyihanguli Maimaiti	Chinese	2014	Anhui	C	HS	3	Yes	U	37	Yes	W	2758	449	16.3
6	2017	Guo Yanwei	Chinese	2013	Hebei	E	HS	3	Yes	27 or less	37	Yes	N	8094	1285	15.9
7	2016	Yang Yinxia	Chinese	2014	Shanxi	C	HS	3	No	U	37	U	W	4903	754	15.4
8	2016	Li Yanhong	Chinese	2012	Guangdong	E	HS	2	No	28	37	Yes	W	8963	1326	14.8
9	2016	Lei Qiong	English	2013	Guangdong	E	HS	3	Yes	27 or less	37	Yes	W	5535	810	14.6
10	2018	Liu Wenyuan	English	2015	Zhejiang	E	HS	3	Yes	27 or less	37	Yes	N	37159	5428	14.6
11	2015	Guo Yeqing	Chinese	2012	Hunan	C	HS	3	Yes	28	37	Yes	W	1031	147	14.3
12	2014	Lei Qiong	Chinese	2011	Guangdong	E	HS	3	Yes	U	37	Yes	W	5498	726	13.2
13	2018	Zhu Hui	English	2016	Fujian	E	HS	3	Yes	U	37	Yes	W	596	76	12.8
14	2017	Zhang Yihui	English	2015	Jiangsu	E	HS	3	No	28	37	No	W	11006	1359	12.3
15	2013	Zhang Jingjing	Chinese	2012	Gansu	W	HS	U	Yes	28	37	Yes	N	1270	154	12.1
16	2018	Huang Huafei	Chinese	2015	Zhejiang	E	HS	3	No	28	37	Yes	N	33958	4090	12.0
17	2017	Xiong Jiangjun	Chinese	2014	Hunan	C	HS	3	No	U	37	U	W	3190	378	11.8

No.	Publication year	First author	Language	Study year	Study setting(s)	Region[a]	Data source[b]	HS level	Singleton only	Lower boundary	Upper boundary	Live birth only	Unit of analysis[c]	Total participants	Number of cases	Rate (%)
18	2017	Qin Jiabi	English	2015	Hunan	C	HS	3	Yes	U	37	No	W	5639	661	11.7
19	2019	Zhao Quming	English	2012	China	China	HS	U	No	U	37	Yes	N	122765	13995	11.4
20	2015	Zhu Li	Chinese	2012	China	China	HS	U	Yes	27 or less	37	Yes	N	159334	17495	11.0
21	2018	Wang Renhong	Chinese	2011	Gansu	W	HS	U	Yes	28	37	No	N	33804	3682	10.9
22	2017	Yan Yan	English	2010	Shanghai	E	HS	3	No	U	37	U	W	1683	183	10.9
23	2013	Fan Lichun	Chinese	2010	Hainan	E	WP	-[e]	No	U	37	Yes	N	89854	9705	10.8
24	2015	Ji Deyong	Chinese	2013	Anhui	C	HS	U	Yes	28	37	Yes	N	6151	664	10.8
25	2012	Liu Lei	Chinese	2010	Anhui	C	HS	3	Yes	U	37	Yes	W	702	74	10.5
26	2015	Xu Haiqing	English	2012	Hubei	C	PS	-	No	U	37	Yes	N	NA[f]	NA	10.5
27	2018	Hou Xiaojing	Chinese	2015	Shanghai	E	HS	3	No	28	37	U	W	600	62	10.3
28	2015	Peng Tingting	Chinese	2013	Shanxi	C	HS	3	Yes	28	37	Yes	N	4804	494	10.3
29	2018	Zhao Xiaoli	Chinese	2012	Guangdong	E	PS	-	No	U	37	Yes	N	786	80	10.2
30	2015	Zhang Xiaobin	Chinese	2012	Xinjiang	W	HS	3	No	28	37	U	W	10880	1100	10.1
31	2012	Yu Yanhua	Chinese	2010	Guangdong	E	HS	3	No	28	37	U	W	2436	244	10.0
32	2016	Liu Xiaohui	English	2011	Gansu	W	HS	3	Yes	27 or less	37	Yes	W	10179	1019	10.0
33	2019	Xu Fengdan	English	2014	China	China	HS	U	No	27 or less	37	Yes	N	138247	13701	9.9
34	2014	Liu Xiyong	Chinese	2012	Zhejiang	E	HS	3	No	28	37	Yes	N	13381	1325	9.9
35	2012	Zhu Yan	Chinese	2011	China	China	HS	U	No	U	37	Yes	N	106078	10498	9.9
36	2016	Zhang Xiaosong	Chinese	2012	Beijing, Guangdong, Hunan, Hubei, Sichuan, Shaanxi	Cross	HS	U	No	27 or less	37	Yes	W	111095	10986	9.9

No.	Publication year	First author	Language	Study year	Study setting(s)	Region[a]	Data source[b]	HS level	Singleton only	Lower boundary	Upper boundary	Live birth only	Unit of analysis[c]	Total participants	Number of cases	Rate (%)
37	2015	Xu Xiaoying	Chinese	2011	Gansu	W	HS	3	Yes	28	37	Yes	W	6181	608	9.8
38	2016	MCT for RDS[g]	Chinese	2013	China	China	HS	U	No	27 or less	37	Yes	N	62880	6017	9.6
39	2018	Han Yingying	English	2015	Jiangsu	E	HS	3	Yes	28	37	Yes	N	6693	638	9.5
40	2019	Chen Chang	English	2016	China	China	HS	U	No	27 or less	37	No	N	75590	7179	9.5
41	2017	Sun Pengfei	English	2013	Hebei	E	WP	-	No	U	37	Yes	N	67718	6213	9.2
42	2016	Jiang Fang	Chinese	2012	Beijing	E	HS	3	No	27 or less	37	U	N	14544	1328	9.1
43	2014	Zhou Xin	Chinese	2011	Jiangsu	E	HS	3	No	28	37	Yes	W	11302	1030	9.1
44	2019	Liu Xijuan	Chinese	2017	Zhejiang	E	HS	3	No	27 or less	37	Yes	N	19800	1800	9.1
45	2017	Li Pengcheng	Chinese	2015	Hubei, Gansu	Cross	HS	U	No	U	37	Yes	W	29712	2662	9.0
46	2018	Yu Honghui	English	2013	Hubei	C	HS	U	No	U	37	No	W	797	71	8.9
47	2013	Tian Qing	Chinese	2011	Guangdong	E	HS	U	No	U	37	Yes	N	34572	3045	8.8
48	2016	Zhong Shilin	Chinese	2012	Guangdong	E	HS	3	No	28	37	Yes	W	19203	1688	8.8
49	2018	Ke Li	English	2015	Guangdong	E	HS	3	Yes	27 or less	37	Yes	W	3684	323	8.8
50	2016	Liu Weiliang	Chinese	2014	Henan, Guangxi, Shanghai	Cross	HS	U	No	28	37	U	W	6000	650	8.8
51	2015	Shen Chongrong	Chinese	2013	Jiangsu	E	HS	2	No	28	37	No	W	1564	136	8.7
52	2015	Fernando C. Barros	English	2011	Beijing	E	HS	U	No	27 or less	37	U	N	6510	547	8.4
53	2016	Zhao Qianqian	Chinese	2012	Beijing	E	HS	3	No	27 or less	37	No	W	11142	930	8.3
54	2014	Zhou J	English	2011	Guangdong	E	HS	3	No	U	37	Yes	W	1953	159	8.1

No.	Publication year	First author	Language	Study year	Study setting(s)	Region[a]	Data source[b]	HS level	Singleton only	Lower boundary	Upper boundary	Live birth only	Unit of analysis[c]	Total participants	Number of cases	Rate (%)
55	2017	Dong Renjing	Chinese	2013	Jilin	NE	HS	3	No	28	37	U	W	14243	1149	8.1
56	2013	Feng Fangfang	Chinese	2011	Hubei	C	HS	3	No	28	37	Yes	W	1400	110	7.9
57	2017	Zhuang Xun	English	2014	Jiangsu	E	HS	3	Yes	27 or less	37	No	W	36755	2875	7.8
58	2017	Liu Yajun	English	2011	Beijing, Jilin, Liaoning, Shanxi, Hebei, Inner Mongolia, Shandong, Shaanxi, Xinjiang	Cross	HS	U	Yes	28	37	Yes	N	65173	5092	7.8
59	2013	Sun Jianhua	Chinese	2011	Liaoning	NE	HS	U	No	28	37	U	W	2370	184	7.8
60	2018	Yan Junmei	Chinese	2013	Jiangsu	E	HS	3	No	27 or less	37	Yes	N	60730	4698	7.7
61	2017	Zeng Lixiang	Chinese	2015	Guangdong	E	HS	3	No	28	37	No	N	34082	2616	7.7
62	2017	Tan Jing	English	2010	Sichuan	W	HS	U	Yes	U	37	U	W	21942	1684	7.7
63[h]	2017	Guo Tongjun	Chinese	2012	Hunan	C	PS	-	Yes	U	37	Yes	W	55654	4212	7.6
64	2015	Chen Ping	Chinese	2013	Zhejiang	E	HS	U	Yes	28	37	U	W	1958	148	7.6
65	2015	Liu Lu	English	2014	Beijing	E	HS	3	Yes	U	37	Yes	W	2973	223	7.5
66	2018	Huang Xin	English	2016	Hunan	C	WP	-	Yes	U	37	Yes	W	16780	1249	7.4
67	2017	Tang Wen	English	2013	China	China	HS	U	No	28	37	Yes	N	3915965	289381	7.4
68	2016	Xu Rong	Chinese	2012	Jiangsu	E	WP	-	Yes	U	37	Yes	W	157187	11530	7.3
69	2012	Lu Rongxian	Chinese	2011	Zhejiang	E	HS	3	No	28	37	Yes	W	3316	243	7.3
70	2018	Wang Xiaojie	Chinese	2017	Guangdong	E	HS	3	No	27 or less	37	Yes	N	6011	439	7.3
71	2017	Wang Shiwen	English	2012	Shandong	E	HS	U	No	U	37	Yes	N	10933	793	7.3

No.	Publication year	First author	Language	Study year	Study setting(s)	Region[a]	Data source[b]	HS level	Singleton only	Lower boundary	Upper boundary	Live birth only	Unit of analysis[c]	Total participants	Number of cases	Rate (%)
72	2013	Yi Lilan	Chinese	2012	Beijing, Heilongjiang, Hunan, Guangdong	Cross	HS	U	Yes	U	37	Yes	N	5539	399	7.2
73	2016	Du Huijuan	Chinese	2013	Hebei	E	HS	2	No	U	37	Yes	N	10644	764	7.2
74	2018	Guo Tongjun	English	2012	China	China	PS	-	Yes	27 or less	37	Yes	N	1020471	73240	7.2
75	2016	Ding Jie	Chinese	2014	Beijing	E	HS	3	Yes	28	37	Yes	W	642	46	7.2
76	2015	Luo Xiaolin	English	2011	China	China	HS	U	No	28	37	No	W	111767	7872	7.0
77	2018	Liu Yiwen	Chinese	2011	Hebei	E	PS	-	Yes	28	37	U	W	993	69	6.9
78	2017	Kang Huixian	Chinese	2012	Beijing	E	HS	3	Yes	28	37	Yes	W	1126	78	6.9
79	2019	Yuan Xiaosong	English	2016	Jiangsu	E	HS	3	Yes	28	37	Yes	W	11576	797	6.9
80	2016	Xue Qinqin	English	2011	Shanghai	E	HS	3	No	27 or less	37	Yes	W	37443	2541	6.8
81	2013	Liu Jingli	Chinese	2012	Jilin	NE	HS	U	No	28	37	U	W	3141	211	6.7
82	2018	Xu Qin	English	2015	China	China	PS	-	No	28	37	No	W	4832887	324207	6.7
83	2013	Ren Dafeng	Chinese	2011	Jiangsu	E	HS	3	No	28	37	U	W	4360	292	6.7
84	2014	Shan Xiaoyi	English	2010	Beijing	E	HS	U	No	U	37	Yes	N	22507	1496	6.6
85	2016	Yu Huiting	Chinese	2015	Shanghai	E	WP	-	No	U	37	Yes	W	191100	12513	6.5
86	2019	Yan Huina	English	2013	Beijing	E	HS	2	Yes	27 or less	37	Yes	W	3688	241	6.5
87	2013	Wang Chengshu	Chinese	2011	Beijing	E	HS	3	Yes	28	37	Yes	W	4090	261	6.4
88	2017	Li Shanshan	Chinese	2016	Guangxi	W	HS	3	No	U	37	Yes	W	1547	98	6.3
89	2017	Xin Qianqian	English	2013	China	China	HS	U	No	U	37	Yes	N	21994	1392	6.3
90	2019	Guan Tianjia	English	2010	Beijing	E	HS	2	No	27 or less	37	Yes	N	82000	5166	6.3

No.	Publication year	First author	Language	Study year	Study setting(s)	Region[a]	Data source[b]	HS level	Singleton only	Lower boundary	Upper boundary	Live birth only	Unit of analysis[c]	Total participants	Number of cases	Rate (%)
91	2014	Guo Yong	English	2012	Guangdong	E	WP	-	Yes	28	37	Yes	W	54575	3384	6.2
92	2018	Ye Lin	English	2014	Jiangsu	E	WP	-	Yes	U	37	Yes	W	24246	1501	6.2
93	2016	Han Shujing	English	2013	Beijing	E	HS	3	No	U	37	Yes	N	37573	2294	6.1
94	2018	Li Changchang	English	2011	Guangdong	E	WP	-	No	27 or less	37	Yes	N	587829	34946	5.9
95	2019	Yang M.	English	2014	Sichuan	W	HS	U	Yes	U	37	Yes	W	4340	252	5.8
96	2016	Zhang Dandan	Chinese	2013	Hubei	C	HS	3	Yes	28	37	Yes	W	11020	630	5.7
97	2015	Yu Rong	Chinese	2012	Beijing	E	HS	3	No	28	37	Yes	W	10678	588	5.5
98	2018	Zhao Jinqi	Chinese	2014	Beijing	E	WP	-	No	U	37	Yes	N	1366377	74994	5.5
99	2019	Su Weijuan	English	2014	Fujian	E	WP	-	No	U	37	No	W	62852	3350	5.3
100	2013	Zheng Feng	Chinese	2011	Liaoning	NE	HS	U	No	28	37	Yes	W	5472	290	5.3
101	2017	Xia Min	English	2012	Shandong	E	HS	3	No	27 or less	37	No	W	10868	571	5.3
102	2018	Xu Jing	English	2016	Shandong	E	HS	3	Yes	U	37	No	W	4552	239	5.3
103	2017	Wang Chen	Chinese	2013	Beijing	E	HS	U	Yes	28	37	Yes	W	14970	785	5.2
104	2018	Li Xiangyu	English	2015	Hubei	C	HS	U	Yes	28	37	Yes	N	10635	557	5.2
105	2019	Wei Liangjia	Chinese	2016	Guangxi	W	HS	U	Yes	28	37	Yes	W	12780	668	5.2
106	2017	Ye Juan	Chinese	2015	Guangxi	W	HS	U	Yes	28	37	U	W	2463	125	5.1
107	2013	Xu Hairong	Chinese	2010	Guangdong	E	HS	2	No	28	37	U	W	1785	90	5.0
108	2019	Lin Xianhua	English	2014	Shanghai	E	HS	3	Yes	28	37	Yes	W	49612	2494	5.0
109	2017	Hu Ronghua	English	2013	Hubei	C	WP	-	Yes	28	37	Yes	W	317463	15889	5.0
110	2015	Bo Sun	Chinese	2012	Shaanxi	W	WP	-	No	U	37	U	N	383976	19198	5.0

No.	Publication year	First author	Language	Study year	Study setting(s)	Region[a]	Data source[b]	HS level	Singleton only	Lower boundary	Upper boundary	Live birth only	Unit of analysis[c]	Total participants	Number of cases	Rate (%)
111	2019	Guo Pi	English	2015	Guangdong	E	WP	-	Yes	U	37	Yes	N	1098600	54521	5.0
112	2019	Liu Tao	Chinese	2017	Guangxi	W	HS	U	No	28	37	U	W	21891	1080	4.9
113	2018	He Jianrong	English	2013	Guangdong	E	HS	3	Yes	U	37	Yes	W	9044	444	4.9
114	2017	Yang Wenjia	English	2012	Beijing	E	HS	3	Yes	U	37	No	W	979	48	4.9
115	2017	Liu Shiping	English	2015	Hunan	C	PS	-	No	U	37	Yes	W	882	43	4.9
116	2018	Xiao Yue	English	2013	Liaoning	NE	HS	U	Yes	U	37	Yes	W	1569	76	4.8
117	2013	Li Guijuan	Chinese	2011	Shandong	E	HS	2	No	U	37	Yes	W	4214	202	4.8
118	2019	Dai Xiaowei	Chinese	2016	Guangdong	E	HS	3	Yes	U	37	Yes	W	1377	66	4.8
119	2018	Wang Chen	English	2014	Beijing, Guangdong, Sichuan	Cross	HS	U	Yes	U	37	U	W	21577	1027	4.8
120	2018	Tang Jiangbing	Chinese	2013	Zhejiang	E	HS	U	No	U	37	U	N	79850	3778	4.7
121	2017	Li Run	English	2013	Sichuan	W	HS	U	Yes	U	37	Yes	W	688	32	4.7
122	2018	Wang Yaocheng	Chinese	2014	Zhejiang	E	WP	-	No	28	37	Yes	N	120819	5532	4.6
123	2015	Pan Lei	English	2011	Tianjin	E	WP	-	Yes	U	37	Yes	W	17545	803	4.6
124	2019	Hu Jie	English	2014	Hubei	C	HS	3	Yes	28	37	Yes	W	16345	721	4.4
125	2018	Xiao Qingyang	English	2012	Shanghai	E	WP	-	Yes	27 or less	37	Yes	N	132783	5856	4.4
126	2014	He Jifei	Chinese	2011	Chongqing	W	HS	U	No	28	37	U	W	8567	366	4.3
127	2015	Xue Xiaoping	Chinese	2010	Shanxi	C	WP	-	Yes	28	37	Yes	N	50871	2163	4.3
128	2014	Xu Xin	Chinese	2011	Hunan	C	HS	U	No	28	37	Yes	W	6270	265	4.2
129	2012	Yang Qing	Chinese	2010	Shanghai	E	HS	2	No	U	37	Yes	N	9741	409	4.2
130	2016	Long Xinzhi	Chinese	2015	Guangdong	E	HS	2	No	28	37	U	W	2000	83	4.2

No.	Publication year	First author	Language	Study year	Study setting(s)	Region[a]	Data source[b]	HS level	Singleton only	Lower boundary	Upper boundary	Live birth only	Unit of analysis[c]	Total participants	Number of cases	Rate (%)
131	2013	Guo Yawei	Chinese	2011	Hunan	C	PS	-	No	28	37	U	W	6270	259	4.1
132	2015	Zhang Cuihong	English	2012	Beijing	E	HS	U	Yes	U	37	Yes	W	14196	586	4.1
133	2019	Zhu Beibei	English	2014	Anhui	C	HS	2	Yes	U	37	Yes	W	3273	134	4.1
134	2018	Ding Haiyan	Chinese	2013	Zhejiang	E	WP	-	No	27 or less	37	No	N	18276	748	4.1
135	2016	Wang Fen	Chinese	2012	Hubei	C	HS	3	No	28	37	Yes	W	24145	986	4.1
136	2019	Ma Haihui	Chinese	2015	Beijing	E	HS	2	Yes	27 or less	37	Yes	W	34726	1418	4.1
137	2015	Zhou Miao	Chinese	2014	Liaoning, Sichuan	Cross	WP	-	No	28	37	Yes	W	7171	289	4.0
138	2018	Tian Jiarong	Chinese	2015	Guangxi	W	WP	-	Yes	28	37	Yes	W	10122	404	4.0
139	2017	Zhao Yanjie	Chinese	2012	Jiangsu	E	WP	-	No	U	37	Yes	N	34022	1339	3.9
140	2018	Wang H.	English	2012	Jiangsu	E	WP	-	No	27 or less	37	No	N	119454	4643	3.9
141	2018	Hu Chengyang	English	2011	Anhui	C	PS	-	No	27 or less	37	Yes	W	16983	656	3.9
142	2018	Zheng Wei	English	2014	Beijing	E	HS	3	Yes	U	37	Yes	N	10366	400	3.9
143	2018	Huang Aiqun	English	2014	Hebei, Liaoning, Hunan, Fujian, Sichuan, Yunnan	Cross	WP	-	Yes	U	37	Yes	W	40152	1331	3.8
144	2014	Fei Lixiao	Chinese	2013	Henan	C	WP	-	No	27 or less	37	U	W	19120	727	3.8
145	2017	Fang Haiqin	Chinese	2015	Jiangsu	E	WP	-	No	28	37	Yes	N	10241	369	3.6
146	2018	Yang Jin	English	2015	Henan	C	HS	U	Yes	U	37	Yes	W	2320	82	3.5
147	2015	Wen Tingyuan	English	2011	Beijing	E	HS	3	Yes	U	37	U	W	13776	485	3.5
148	2015	Jia Xiaomin	Chinese	2011	Anhui	C	HS	3	Yes	28	37	Yes	W	2729	96	3.5
149	2014	Chen Liangmiao	English	2010	Zhejiang	E	HS	3	No	28	37	No	W	8012	281	3.5

No.	Publication year	First author	Language	Study year	Study setting(s)	Region[a]	Data source[b]	HS level	Singleton only	Lower boundary	Upper boundary	Live birth only	Unit of analysis[c]	Total participants	Number of cases	Rate (%)
150	2017	Fan Xiaojing	English	2011	Tibet	W	PS	-	Yes	28	37	Yes	W	701	23	3.3
151	2019	Fang Jing	English	2014	Hubei	C	HS	3	Yes	U	37	Yes	W	1028	33	3.2
152	2014	Li Yanhua	Chinese	2010	Fujian	E	HS	3	Yes	U	37	Yes	W	592	19	3.2
153	2019	Li Minmin	Chinese	2012	Shaanxi	W	PS	-	Yes	U	37	Yes	W	28841	894	3.1
154	2016	Hu Yan	Chinese	2013	Hubei	C	HS	3	No	28	37	Yes	W	5242	160	3.1
155	2016	Jin Wenyuan	English	2011	Zhejiang	E	HS	3	Yes	28	37	Yes	W	934	27	2.9
156	2018	Zhao Xiang	Chinese	2017	Shandong	E	HS	3	Yes	U	37	Yes	W	1300	37	2.8
157[h]	2017	Guo Tongjun	English	2014	Henan	C	PS	-	Yes	U	37	U	N	244305	6709	2.7
158	2016	Guo Yanwei	Chinese	2010	Hebei	E	HS	U	No	28	37	No	W	4966	131	2.6
159	2014	Wang Jing	Chinese	2012	Beijing	E	HS	2	No	U	37	U	W	4042	103	2.5
160	2016	Jiang Yan	Chinese	2010	Guangdong	E	HS	2	No	U	37	U	W	5000	120	2.4
161	2013	Wang Mei	Chinese	2011	Ningxia	W	HS	2	Yes	U	37	Yes	W	600	13	2.2
162	2019	Wang Feixue	Chinese	2016	Zhejiang	E	PS	-	No	U	37	Yes	W	520695	8151	1.6
CR[i] (%)	99.4	95.7	100.0	90.7	96.9	-	91.4	-	96.9	73.5	92.6	73.5	86.4	84.0	87.0	79.0

[a] "NE": Northeastern China; "E": Eastern China; "C": Central China; "W": Western China.

[b] "WP": Whole population; "PS": Population sample; "HS": Hospital.

[c] "N": Neonate; "W": Woman.

[d] "U": Unknown. Hospital level of hospital-based studies was unknown if: 1) it could not be determined according to the online system, 2) there was no specific hospital name provided, 3) hospitals were of different types.

[e] "-": Not applicable, including: 1) hospital level for studies from whole population or population sample; 2) consistency rates for region and hospital level, as they were not directly extracted from studies.

[f] Data were unobtainable.

[g] "MCT for RDS": Multicenter Collaborative Team for the Respiratory Distress Syndrome.

[h] See eText2 by No. for details on two studies with same publication year and first author.

[i] "CR": Consistency rate (of data extraction between two reviewers), the percentage of studies from which the data extracted by the two reviewers were consistent relative to the total number of studies included.

eTable3. Study quality assessment (n=162)

No.	Publication year	First author	The 9 domains of Joanna Briggs Institute Critical Appraisal Checklist								
			Sample frame[a]	Sampling method[b]	Sample size[c]	Subject and setting description[d]	Identified sample coverage[e]	Identification method[f]	Measurement[g]	Statistical analysis[h]	Response rate[i]
1	2013	Hu Chunxia	No	Yes	No	No	Yes	Yes	Yes	Yes	Yes
2	2015	Zhu Tong	No	Yes	No	Yes	Unclear	Yes	Yes	Yes	No
3	2014	Kong Dechuan	No	Yes	No	Yes	Yes	Yes	Yes	Yes	Yes
4	2013	Zhou Shenglan	No	Yes	Yes	Yes	Unclear	Yes	Yes	Yes	Unclear
5	2017	Reyihanguli Maimaiti	No	Yes	Yes	Yes	Yes	Yes	Yes	Yes	Yes
6	2017	Guo Yanwei	No	Yes	Yes	Yes	Yes	Yes	Yes	Yes	Yes
7	2016	Yang Yinxia	No	Yes	Yes	No	Yes	Yes	Yes	Yes	Yes
8	2016	Li Yanhong	No	Yes	Yes	No	Yes	Yes	Yes	Yes	Yes
9	2016	Lei Qiong	No	Yes	Yes	Yes	Yes	Yes	Yes	Yes	Yes
10	2018	Liu Wenyuan	No	Yes	Yes	Yes	Yes	Yes	Yes	Yes	Yes
11	2015	Guo Yeqing	No	Yes	No	Yes	Yes	Yes	Yes	Yes	Yes
12	2014	Lei Qiong	No	Yes	Yes	Yes	Yes	Yes	Yes	Yes	Yes
13	2018	Zhu Hui	No	Yes	No	Yes	Yes	Yes	Yes	Yes	Yes
14	2017	Zhang Yihui	No	Yes	Yes	Yes	Yes	Yes	Yes	Yes	Yes
15	2013	Zhang Jingjing	No	Yes	No	Yes	Unclear	Yes	Yes	Yes	Unclear
16	2018	Huang Huafei	No	Yes	Yes	No	Yes	Yes	Yes	Yes	Yes
17	2017	Xiong Jiangjun	No	Yes	Yes	No	Yes	Yes	Yes	Yes	Yes
18	2017	Qin Jiabi	No	Yes	Yes	Yes	Unclear	Yes	Yes	Yes	No

No.	Publication year	First author	The 9 domains of Joanna Briggs Institute Critical Appraisal Checklist								
			Sample frame[a]	Sampling method[b]	Sample size[c]	Subject and setting description[d]	Identified sample coverage[e]	Identification method[f]	Measurement[g]	Statistical analysis[h]	Response rate[i]
19	2019	Zhao Quming	Yes	No	Yes	Yes	Yes	Yes	Yes	Yes	Yes
20	2015	Zhu Li	Yes	No	Yes	Yes	Yes	Yes	Yes	Yes	Yes
21	2018	Wang Renhong	No	Yes	Yes	Yes	Yes	Yes	Yes	Yes	Yes
22	2017	Yan Yan	No	Yes	No	Yes	Unclear	Yes	Yes	Yes	Unclear
23	2013	Fan Lichun	Yes	Yes	Yes	No	Yes	Yes	Yes	Yes	Yes
24	2015	Ji Deyong	No	Yes	Yes	Yes	Yes	Yes	Yes	Yes	Yes
25	2012	Liu Lei	No	Yes	No	Yes	Yes	Yes	Yes	Yes	Yes
26	2015	Xu Haiqing	Yes	Yes	Yes	Yes	Yes	Yes	Yes	Yes	Yes
27	2018	Hou Xiaojing	No	Yes	No	Yes	Yes	Yes	Yes	Yes	Yes
28	2015	Peng Tingting	No	Yes	Yes	Yes	Yes	Yes	Yes	Yes	Yes
29	2018	Zhao Xiaoli	Yes	No	No	Yes	Unclear	Yes	Yes	Yes	Unclear
30	2015	Zhang Xiaobin	No	Yes	Yes	No	Yes	Yes	Yes	Yes	Yes
31	2012	Yu Yanhua	No	Yes	Yes	Yes	Yes	Yes	Yes	Yes	Yes
32	2016	Liu Xiaohui	No	Yes	Yes	Yes	Unclear	Yes	Yes	Yes	No
33	2019	Xu Fengdan	Yes	No	Yes	Yes	Yes	Yes	Yes	Yes	Yes
34	2014	Liu Xiyong	No	Yes	Yes	Yes	Yes	Yes	Yes	Yes	Yes
35	2012	Zhu Yan	Yes	No	Yes	Yes	Yes	Yes	Yes	Yes	Yes
36	2016	Zhang Xiaosong	No	No	Yes	Yes	Yes	Yes	Yes	Yes	Yes
37	2015	Xu Xiaoying	No	Yes	Yes	Yes	Yes	Yes	Yes	Yes	Yes

No.	Publication year	First author	The 9 domains of Joanna Briggs Institute Critical Appraisal Checklist								
			Sample frame[a]	Sampling method[b]	Sample size[c]	Subject and setting description[d]	Identified sample coverage[e]	Identification method[f]	Measurement[g]	Statistical analysis[h]	Response rate[i]
38	2016	MCT for RDS	No	Yes	Yes	Yes	Yes	Yes	Yes	Yes	Yes
39	2018	Han Yingying	No	Yes	Yes	Yes	Yes	Yes	Yes	Yes	Yes
40	2019	Chen Chang	No	No	Yes	Yes	Yes	Yes	Yes	Yes	Yes
41	2017	Sun Pengfei	Yes	Yes	Yes	No	Yes	Yes	Yes	Yes	Yes
42	2016	Jiang Fang	No	Yes	Yes	Yes	Yes	Yes	Yes	Yes	Yes
43	2014	Zhou Xin	No	Yes	Yes	No	Yes	Yes	Yes	Yes	Yes
44	2019	Liu Xijuan	No	Yes	Yes	Yes	Yes	Yes	Yes	Yes	Yes
45	2017	Li Pengcheng	No	No	Yes	Yes	Yes	Yes	Yes	Yes	Yes
46	2018	Yu Honghui	No	Yes	No	Yes	Yes	Yes	Yes	Yes	Yes
47	2013	Tian Qing	No	Yes	Yes	Yes	Yes	Yes	Yes	Yes	Yes
48	2016	Zhong Shilin	No	Yes	Yes	No	Yes	Yes	Yes	Yes	Yes
49	2018	Ke Li	No	Yes	Yes	Yes	Yes	Yes	Yes	Yes	Yes
50	2016	Liu Weiliang	No	Unclear	Yes	No	Yes	Yes	Yes	Yes	Yes
51	2015	Shen Chongrong	No	Yes	No	Yes	Yes	Yes	Yes	Yes	Yes
52	2015	Fernando C. Barros	No	Yes	Yes	Yes	Yes	Yes	Yes	Yes	Yes
53	2016	Zhao Qianqian	No	Yes	Yes	Yes	Yes	Yes	Yes	Yes	Yes
54	2014	Zhou J	No	Yes	No	Yes	Unclear	Yes	Yes	Yes	No
55	2017	Dong Renjing	No	Yes	Yes	Yes	No	Yes	Yes	Yes	Yes
56	2013	Feng Fangfang	No	Yes	No	No	Yes	Yes	Yes	Yes	Yes

No.	Publication year	First author	The 9 domains of Joanna Briggs Institute Critical Appraisal Checklist								
			Sample frame[a]	Sampling method[b]	Sample size[c]	Subject and setting description[d]	Identified sample coverage[e]	Identification method[f]	Measurement[g]	Statistical analysis[h]	Response rate[i]
57	2017	Zhuang Xun	No	Yes	Yes	Yes	Yes	Yes	Yes	Yes	Yes
58	2017	Liu Yajun	Yes	No	Yes	No	Yes	Yes	Yes	Yes	Yes
59	2013	Sun Jianhua	No	Yes	Yes	No	Yes	Yes	Yes	Yes	Yes
60	2018	Yan Junmei	No	Yes	Yes	Yes	Yes	Yes	Yes	Yes	Yes
61	2017	Zeng Lixiang	No	Yes	Yes	Yes	Yes	Yes	Yes	Yes	Yes
62	2017	Tan Jing	No	Yes	Yes	Yes	Yes	Yes	Yes	Yes	Yes
63[j]	2017	Guo Tongjun	Yes	Unclear	Yes	Yes	Unclear	Yes	Yes	Yes	Unclear
64	2015	Chen Ping	No	Yes	No	Yes	Unclear	Yes	Yes	Yes	Unclear
65	2015	Liu Lu	No	Yes	Yes	Yes	Yes	Yes	Yes	Yes	Yes
66	2018	Huang Xin	Yes	Yes	Yes	Yes	Yes	Yes	Yes	Yes	Yes
67	2017	Tang Wen	Yes	No	Yes	Yes	No	Yes	Yes	Yes	Yes
68	2016	Xu Rong	Yes	Yes	Yes	Yes	Yes	Yes	Yes	Yes	Yes
69	2012	Lu Rongxian	No	Yes	Yes	Yes	Yes	Yes	Yes	Yes	Yes
70	2018	Wang Xiaojie	No	Yes	Yes	Yes	Yes	Yes	Yes	Yes	Yes
71	2017	Wang Shiwen	Yes	No	Yes	Yes	Yes	Yes	Yes	Yes	Yes
72	2013	Yi Lilan	No	Yes	Yes	Yes	Yes	Yes	Yes	Yes	Yes
73	2016	Du Huijuan	No	Yes	Yes	Yes	Yes	Yes	Yes	Yes	Yes
74	2018	Guo Tongjun	Yes	No	Yes	Yes	No	Yes	Yes	Yes	Yes
75	2016	Ding Jie	No	Yes	No	Yes	Unclear	Yes	Yes	Yes	No

No.	Publication year	First author	The 9 domains of Joanna Briggs Institute Critical Appraisal Checklist								
			Sample frame[a]	Sampling method[b]	Sample size[c]	Subject and setting description[d]	Identified sample coverage[e]	Identification method[f]	Measurement[g]	Statistical analysis[h]	Response rate[i]
76	2015	Luo Xiaolin	Yes	No	Yes	Yes	Yes	Yes	Yes	Yes	Yes
77	2018	Liu Yiwen	No	Yes	No	Yes	No	Yes	Yes	Yes	Yes
78	2017	Kang Huixian	No	No	No	Yes	Unclear	Yes	Yes	Yes	Yes
79	2019	Yuan Xiaosong	No	Yes	Yes	Yes	Yes	Yes	Yes	Yes	Yes
80	2016	Xue Qinqin	No	Yes	Yes	Yes	Yes	Yes	Yes	Yes	Yes
81	2013	Liu Jingli	No	Yes	Yes	No	Yes	Yes	Yes	Yes	Yes
82	2018	Xu Qin	Yes	No	Yes	No	No	Yes	Yes	Yes	Yes
83	2013	Ren Dafeng	No	Yes	Yes	Yes	Yes	Yes	Yes	Yes	Yes
84	2014	Shan Xiaoyi	No	Yes	Yes	Yes	Yes	Yes	Yes	Yes	Yes
85	2016	Yu Huiting	Yes	Yes	Yes	No	Yes	Yes	Yes	Yes	Yes
86	2019	Yan Huina	No	Yes	Yes	Yes	Yes	Yes	Yes	Yes	Yes
87	2013	Wang Chengshu	No	Yes	Yes	Yes	Yes	Yes	Yes	Yes	Yes
88	2017	Li Shanshan	No	Yes	No	Yes	Unclear	Yes	Unclear	Yes	No
89	2017	Xin Qianqian	Yes	No	Yes	Yes	Unclear	Yes	Yes	Yes	Unclear
90	2019	Guan Tianjia	No	Yes	Yes	No	Yes	Yes	Yes	Yes	Yes
91	2014	Guo Yong	Yes	Yes	Yes	Yes	Yes	Yes	Yes	Yes	Yes
92	2018	Ye Lin	Yes	Yes	Yes	Yes	Yes	Yes	Yes	Yes	Yes
93	2016	Han Shujing	No	Yes	Yes	Yes	Yes	Yes	Yes	Yes	Yes
94	2018	Li Changchang	Yes	Yes	Yes	Yes	Yes	Yes	Yes	Yes	Yes

No.	Publication year	First author	The 9 domains of Joanna Briggs Institute Critical Appraisal Checklist								
			Sample frame[a]	Sampling method[b]	Sample size[c]	Subject and setting description[d]	Identified sample coverage[e]	Identification method[f]	Measurement[g]	Statistical analysis[h]	Response rate[i]
95	2019	Yang M.	Yes	No	Yes	Yes	Yes	Yes	Yes	Yes	Yes
96	2016	Zhang Dandan	No	Yes	Yes	Yes	Yes	Yes	Yes	Yes	Yes
97	2015	Yu Rong	No	Yes	Yes	No	Yes	Yes	Yes	Yes	Yes
98	2018	Zhao Jinqi	Yes	Yes	Yes	Yes	Yes	Yes	Yes	Yes	Yes
99	2019	Su Weijuan	Yes	Yes	Yes	Yes	Yes	Yes	Yes	Yes	Yes
100	2013	Zheng Feng	No	Yes	Yes	Yes	Yes	Yes	Yes	Yes	Yes
101	2017	Xia Min	No	Yes	Yes	Yes	Yes	Yes	Yes	Yes	Yes
102	2018	Xu Jing	No	Yes	Yes	No	Yes	Yes	Yes	Yes	Yes
103	2017	Wang Chen	Yes	No	Yes	Yes	Yes	Yes	Yes	Yes	Yes
104	2018	Li Xiangyu	No	Yes	Yes	Yes	Yes	Yes	Yes	Yes	Yes
105	2019	Wei Liangjia	No	Yes	Yes	Yes	Yes	Yes	Yes	Yes	Yes
106	2017	Ye Juan	No	Yes	Yes	Yes	Yes	Yes	Yes	Yes	Yes
107	2013	Xu Hairong	No	Yes	No	No	Yes	Yes	Yes	Yes	Yes
108	2019	Lin Xianhua	No	Yes	Yes	Yes	Yes	Yes	Yes	Yes	Yes
109	2017	Hu Ronghua	Yes	Yes	Yes	Yes	Yes	Yes	Yes	Yes	No
110	2015	Bo Sun	Yes	Yes	Yes	No	Yes	Yes	Yes	Yes	Yes
111	2019	Guo Pi	Yes	Yes	Yes	Yes	Yes	Yes	Yes	Yes	Yes
112	2019	Liu Tao	No	Yes	Yes	Yes	Unclear	Yes	Yes	Yes	Unclear
113	2018	He Jianrong	No	Yes	Yes	Yes	Yes	Yes	Yes	Yes	Yes

No.	Publication year	First author	The 9 domains of Joanna Briggs Institute Critical Appraisal Checklist								
			Sample frame[a]	Sampling method[b]	Sample size[c]	Subject and setting description[d]	Identified sample coverage[e]	Identification method[f]	Measurement[g]	Statistical analysis[h]	Response rate[i]
114	2017	Yang Wenjia	No	Yes	No	Yes	Unclear	Yes	Yes	Yes	Unclear
115	2017	Liu Shiping	Yes	Unclear	No	Yes	Yes	Yes	No	Yes	Yes
116	2018	Xiao Yue	No	Yes	No	No	Yes	Yes	Yes	Yes	Yes
117	2013	Li Guijuan	No	Yes	Yes	Yes	Yes	Yes	Yes	Yes	Yes
118	2019	Dai Xiaowei	No	Yes	No	Yes	Unclear	Yes	Yes	Yes	Unclear
119	2018	Wang Chen	No	Yes	Yes	Yes	Yes	Yes	Yes	Yes	Yes
120	2018	Tang Jiangbing	No	Yes	Yes	Yes	Yes	Yes	Yes	Yes	Yes
121	2017	Li Run	No	Yes	No	Yes	Yes	Yes	Yes	Yes	Yes
122	2018	Wang Yaocheng	Yes	Yes	Yes	Yes	Yes	Yes	Yes	Yes	Yes
123	2015	Pan Lei	Yes	Yes	Yes	Yes	Yes	Yes	Yes	Yes	Yes
124	2019	Hu Jie	No	Yes	Yes	Yes	No	Yes	Yes	Yes	No
125	2018	Xiao Qingyang	Yes	Yes	Yes	Yes	Yes	Yes	Yes	Yes	Yes
126	2014	He Jifei	No	Yes	Yes	No	Yes	Yes	Yes	Yes	Yes
127	2015	Xue Xiaoping	Yes	Yes	Yes	Yes	Yes	Yes	Yes	Yes	Yes
128	2014	Xu Xin	Yes	Yes	Yes	Yes	Yes	Yes	Yes	Yes	Yes
129	2012	Yang Qing	No	Yes	Yes	Yes	Yes	Yes	Yes	Yes	Yes
130	2016	Long Xinzhi	No	Yes	Yes	No	Unclear	Yes	Yes	Yes	Unclear
131	2013	Guo Yawei	No	Yes	Yes	Yes	No	Yes	Yes	Yes	Yes
132	2015	Zhang Cuihong	No	Yes	Yes	Yes	Yes	Yes	Yes	Yes	Yes

No.	Publication year	First author	The 9 domains of Joanna Briggs Institute Critical Appraisal Checklist								
			Sample frame[a]	Sampling method[b]	Sample size[c]	Subject and setting description[d]	Identified sample coverage[e]	Identification method[f]	Measurement[g]	Statistical analysis[h]	Response rate[i]
133	2019	Zhu Beibei	No	Yes	Yes	Yes	Yes	Yes	Yes	Yes	Yes
134	2018	Ding Haiyan	Yes	Yes	Yes	Yes	Yes	Yes	Yes	Yes	Yes
135	2016	Wang Fen	No	Yes	Yes	Yes	Yes	Yes	Yes	Yes	Yes
136	2019	Ma Haihui	No	Yes	Yes	Yes	Unclear	Yes	Yes	Yes	No
137	2015	Zhou Miao	Yes	Yes	Yes	Yes	Yes	Yes	Yes	Yes	Yes
138	2018	Tian Jiarong	Yes	Yes	Yes	Yes	Yes	Yes	Yes	Yes	Yes
139	2017	Zhao Yanjie	Yes	Yes	Yes	Yes	Yes	Yes	Yes	Yes	Yes
140	2018	Wang H.	Yes	Yes	Yes	Yes	Yes	Yes	Yes	Yes	Yes
141	2018	Hu Chengyang	Yes	Yes	Yes	Yes	No	Yes	Yes	Yes	Yes
142	2018	Zheng Wei	No	Yes	Yes	Yes	Yes	Yes	Yes	Yes	Yes
143	2018	Huang Aiqun	Yes	Yes	Yes	Yes	Yes	Yes	Yes	Yes	Yes
144	2014	Fei Lixiao	Yes	Yes	Yes	No	Yes	Yes	Yes	Yes	Yes
145	2017	Fang Haiqin	Yes	Yes	Yes	Yes	Yes	Yes	Yes	Yes	Yes
146	2018	Yang Jin	No	No	Yes	Yes	Unclear	Yes	Yes	Yes	No
147	2015	Wen Tingyuan	No	Yes	Yes	Yes	Unclear	Yes	Yes	Yes	Unclear
148	2015	Jia Xiaomin	No	Yes	Yes	Yes	Unclear	Yes	Yes	Yes	No
149	2014	Chen Liangmiao	No	Yes	Yes	Yes	Unclear	Yes	Yes	Yes	Unclear
150	2017	Fan Xiaojing	No	Yes	No	Yes	No	Yes	Yes	Yes	Yes
151	2019	Fang Jing	No	Yes	No	Yes	Yes	Yes	Yes	Yes	Yes

No.	Publication year	First author	The 9 domains of Joanna Briggs Institute Critical Appraisal Checklist								
			Sample frame[a]	Sampling method[b]	Sample size[c]	Subject and setting description[d]	Identified sample coverage[e]	Identification method[f]	Measurement[g]	Statistical analysis[h]	Response rate[i]
152	2014	Li Yanhua	No	Yes	No	Yes	Yes	Yes	Yes	Yes	Yes
153	2019	Li Minmin	Yes	Yes	Yes	Yes	Yes	Yes	Yes	Yes	Yes
154	2016	Hu Yan	No	Yes	Yes	Yes	Yes	Yes	Yes	Yes	Yes
155	2016	Jin Wenyuan	No	Yes	No	Yes	Yes	Yes	Yes	Yes	Yes
156	2018	Zhao Xiang	No	Yes	No	Yes	Yes	Yes	Yes	Yes	Yes
157[j]	2017	Guo Tongjun	Yes	Unclear	Yes	No	Unclear	Yes	Yes	Yes	Unclear
158	2016	Guo Yanwei	No	Yes	Yes	Yes	Yes	Yes	Yes	Yes	Yes
159	2014	Wang Jing	No	Yes	Yes	No	Yes	Yes	Yes	Yes	Yes
160	2016	Jiang Yan	No	Yes	Yes	Yes	Yes	Yes	Yes	Yes	Yes
161	2013	Wang Mei	No	Unclear	No	No	No	Yes	Yes	Yes	Unclear
162	2019	Wang Feixue	Yes	No	Yes	No	No	Yes	Yes	Yes	Yes
Consistency rate (%)			84.0	80.9	96.9	72.2	75.3	92.6	94.4	932	80.3

[a] The target population should be addressed appropriately with the sample frame. “Yes” for the census, studies with individual sample from whole population, and studies with cluster sample from all hospitals.

[b] Study participants should be recruited in an appropriate way. “Yes” for the census and studies with random samples.

[c] The sample size should be adequate. “Yes” for sample size≥2000.

[d] The study subjects and setting should be described in detail. “Yes” for studies with sufficient details on subjects and settings.

[e] Data analysis should be conducted with sufficient coverage of the identified sample. “Yes” for same response rate among different subgroup sample.

[f] Valid methods should be used for the identification of the condition. “Yes” for studies using an upper boundary of 37 gestational weeks to define preterm delivery.

[g] The condition should be measured in a standard, reliable way for all participants. “Yes” for population-based studies with reliable measurement of gestational weeks and hospital-based studies.

[h] Statistical analysis should be appropriate. “Yes” for studies from which the numerator and denominator of preterm delivery can be directly obtained.

[i] The response rate should be adequate, and if not, the low response rate should be managed appropriately. “Yes” for studies with response rate not less than 85%.

[j] See eText2 by No. for details on two studies with same publication year and first author.

eTable4. Stratified analysis within population-based studies (n=37)

	Number of records	Participants	Rate (%, 95% CI)	I^2 (%, 95% CI); $P_{heterogeneity}$[a]	$P_{between\text{-}subgroup}$[a]
**Total studies**	37	11728601	4.9 (4.5, 5.4)	99.91 (99.90, 99.91); <0.001	-
**Language**					
Chinese	19	3072375	4.8 (4.0, 5.9)	99.92 (99.91, 99.92); <0.001	0.621
English	18	8656226	5.1 (4.6, 5.6)	99.89 (99.88, 99.90); <0.001	
**Study year**					
2010	2	140725	6.8 (2.7, 16.9)	99.94 (99.92, 99.95); <0.001	0.747
2011	6	630321	4.7 (3.8, 5.8)	97.73 (96.58, 98.50); <0.001	
2012	10	1987749	5.5 (4.7, 6.4)	99.84 (99.82, 99.86); <0.001	
2013	4	422577	5.2 (3.5, 7.7)	99.85 (99.82, 99.88); <0.001	
2014	7	1865922	4.4 (3.4, 5.5)	99.83 (99.80, 99.85); <0.001	
2015	6	6143832	5.0 (4.3, 5.9)	99.89 (99.88, 99.91); <0.001	
2016	2	537475	3.4 (0.7, 15.7)	99.96 (99.96, 99.97); <0.001	
**Singleton only**					
Yes	17	3271289	5.0 (4.3, 5.7)	99.87 (99.85, 99.88); <0.001	0.927
No	20	8457312	4.9 (4.3, 5.6)	99.93 (99.93, 99.93); <0.001	
**Lower boundary**					
28	11	5412113	4.7 (4.1, 5.4)	99.66 (99.61, 99.71); <0.001	0.868
27 or less	7	1914916	4.6 (3.9, 5.5)	99.84 (99.82, 99.86); <0.001	
**Live birth only**					

	Number of records	Participants	Rate (%, 95% CI)	I^2 (%, 95% CI); $P_{heterogeneity}$[a]	$P_{between\text{-}subgroup}$[a]
Yes	28	6040468	5.1 (4.5, 5.7)	99.91 (99.90, 99.91); <0.001	0.812
No	4	5033469	4.9 (3.6, 6.7)	99.83 (99.79, 99.86); <0.001	
**Unit of analysis**					
Woman	21	6382219	4.7 (4.0, 5.6)	99.90 (99.90, 99.91); <0.001	0.396
Neonate	16	5346382	5.2 (4.6, 5.9)	99.91 (99.91, 99.92); <0.001	
**Region**					
Total studies	34[b]	5837273	4.9 (4.4, 5.5)	99.88 (99.88, 99.89); <0.001	-
Eastern China	20	4675952	5.3 (4.6, 6.2)	99.92 (99.91, 99.92); <0.001	0.066
Central China	9	728328	4.6 (3.6, 6.0)	99.77 (99.73, 99.80); <0.001	
Western China	5	432993	3.8 (2.9, 4.9)	98.46 (97.68, 98.97); <0.001	
**Sample Size**					
Yes	33	11725239	4.9 (4.4, 5.3)	99.92 (99.92, 99.92); <0.001	0.377
No	4	3362	5.9 (3.8, 9.2)	90.64 (79.07, 95.81); <0.001	
**Identified sample coverage**					
Yes	27	5028856	5.0 (4.6, 5.5)	99.78 (99.76, 99.80); <0.001	0.294
No	7	6399000	4.3 (3.3, 5.7)	99.97 (99.96, 99.97); <0.001	
**Response rate**					
Yes	33	11110393	4.9 (4.5, 5.3)	99.91 (99.90, 99.91); <0.001	0.573
No	1	317463	5.0 (4.9, 5.1)	-	

[a] Tested by Cochrane Q.

[b] Studies covered more than one region without obtainable region-specific data were not included in region stratified analysis.

eTable5. Stratified analysis within hospital-based studies (n=124)

	Number of records	Participants	Rate (%, 95% CI)	I^2 (%, 95% CI); $P_{heterogeneity}$[a]	$P_{between\text{-}subgroup}$[a]
**Total studies**	124	6171419	7.2 (6.9, 7.6)	99.53 (99.50, 99.55); <0.001	-
**Study year**					
2010	12	161366	5.5 (4.6, 6.5)	98.21 (97.69, 98.62); <0.001	0.006
2011	27	496240	7.4 (6.5, 8.3)	99.36 (99.28, 99.43); <0.001	
2012	23	561104	8.4 (7.5, 9.3)	99.32 (99.22, 99.40); <0.001	
2013	22	4279003	7.2 (6.5, 8.0)	99.36 (99.26, 99.44); <0.001	
2014	15	302009	7.0 (5.7, 8.6)	99.51 (99.43, 99.57); <0.001	
2015	14	214677	7.7 (6.2, 9.5)	99.57 (99.50, 99.63); <0.001	
2016	7	108018	6.8 (5.3, 8.8)	98.51 (97.93, 98.93); <0.001	
2017	4	49002	5.7 (3.9, 8.3)	99.01 (98.52, 99.34); <0.001	
**Singleton only**					
Yes	58	704214	6.9 (6.2, 7.7)	99.41 (99.37, 99.46); <0.001	0.284
No	66	5467205	7.4 (7.0, 7.9)	99.56 (99.53, 99.59); <0.001	
**Lower boundary**					
28	55	4582634	7.2 (6.7, 7.8)	99.36 (99.31, 99.41); <0.001	<0.001
27 or less	24	937863	9.4 (8.4, 10.3)	99.58 (99.53, 99.62); <0.001	
**Live birth only**					
Yes	82	5552503	7.2 (6.8, 7.7)	99.57 (99.55, 99.60); <0.001	0.413
No	16	353142	7.8 (6.6, 9.1)	99.43 (99.33, 99.51); <0.001	
**Unit of analysis**					

	Number of records	Participants	Rate (%, 95% CI)	I^2 (%, 95% CI); $P_{heterogeneity}$[a]	$P_{between\text{-}subgroup}$[a]
Woman	88	901221	6.7 (6.1, 7.3)	99.24 (99.18, 99.29); <0.001	<0.001
Neonate	36	5270198	8.5 (7.9, 9.3)	99.76 (99.74, 99.78); <0.001	
**Hospital level**					
Tertiary	68	708531	8.0 (7.2, 8.8)	99.37 (99.32, 99.42); <0.001	0.001
Secondary	14	172240	4.9 (3.8, 6.3)	99.27 (99.13, 99.39); <0.001	
**Region**					
Total studies	134[b]	1329755	7.4 (6.9, 7.9)	99.33 (99.29, 99.37); <0.001	-
Northeastern China	8	35147	8.7 (5.8, 13.0)	99.24 (99.03, 99.40); <0.001	0.703
Eastern China	75	984762	7.1 (6.5, 7.8)	99.46 (99.43, 99.50); <0.001	
Central China	26	129146	7.6 (6.1, 9.5)	99.21 (99.09, 99.30); <0.001	
Western China	25	180700	7.6 (6.6, 8.7)	98.59 (98.34, 98.80); <0.001	

[a] Tested by Cochrane Q.

[b] Studies covered more than one region without obtainable region-specific data were not included in region stratified analysis.

eTable6. Province-level pooled rates and nationwide weighted rate

Study setting	Number of records[a]	Participants	Rate (%, 95% CI)[b]	Number of live births[c]	Estimated number of preterm deliveries
Anhui	1	16983	3.9 (3.6, 4.2)	6638456	256423
Beijing	1	1366377	5.5 (5.5, 5.5)	1358237	74547
Fujian	1	62852	5.3 (5.2, 5.5)	4581584	244198
Guangdong	4	1741790	6.3 (5.5, 7.2)	12798165	800819
Guangxi	1	10122	4.0 (3.6, 4.4)	7112152	283868
Hainan	1	89854	10.8 (10.6, 11.0)	1058478	114325
Hebei	2	68711	8.2 (6.3, 10.7)	8604691	703731
Hebei, Liaoning	1	16691	3.8 (3.6, 4.1)	2716689[d]	104169
Henan	2	263425	3.2 (2.3, 4.4)	13354764	430761
Hubei	1	317463	5.0 (4.9, 5.1)	5698488	285209
Hunan	4	79586	6.0 (4.9, 7.3)	7180633	427266
Jiangsu	5	345150	4.8 (3.4, 6.7)	6744659	322902
Shaanxi	2	412817	3.9 (2.5, 6.3)	3525820	138954
Shanghai	2	323883	5.4 (3.6, 7.9)	1039668	55876
Shanxi	1	50871	4.3 (4.1, 4.4)	3153449	134082
Sichuan, Yunnan	1	9353	3.5 (3.2, 3.9)	12156002[e]	426298
Tianjin	1	17545	4.6 (4.3, 4.9)	864963	39588
Tibet	1	701	3.3 (2.2, 4.9)	411515	13502
Zhejiang	3	659790	3.1 (1.4, 7.0)	4297752	132518

Sum	103296165	4989035
**Weighted rate (%)**	**4.8 (4.0, 5.9)**	

[a] Studies with nationwide samples were not included as their province-level data were unobtainable.

[b] Rates for settings with records≥2 were pooled using the random-effects model, and records in each corresponding group were highly heterogeneous with I^2s ranging from 82.35% to 99.95%.

[c] Accumulated from 2010 to 2018 according to China Health Statistical Yearbook.

[d] Only data of Liaoning were used since a study purely from Hebei was already included.

[e] Including data from Sichuan and Yunnan.

Study	Total participants	Cases	Cases per 100 participants	Rate (%)	95% CI	Weight
Hu Chunxia 2013	1619	470		29.0	[26.8; 31.3]	0.6%
Zhu Tong 2015	821	180		21.9	[19.1; 24.9]	0.6%
Kong Dechuan 2014	1028	219		21.3	[18.8; 23.9]	0.6%
Zhou Shenglan 2013	6063	1123		18.5	[17.6; 19.5]	0.6%
Reyihanguli Maimaiti 2017	2758	449		16.3	[14.9; 17.7]	0.6%
Guo Yanwei 2017	8094	1285		15.9	[15.1; 16.7]	0.6%
Yang Yinxia 2016	4903	754		15.4	[14.4; 16.4]	0.6%
Li Yanhong 2016	8963	1326		14.8	[14.1; 15.5]	0.6%
Lei Qiong 2016	5535	810		14.6	[13.7; 15.6]	0.6%
Liu Wenyuan 2018	37159	5428		14.6	[14.2; 15.0]	0.7%
Guo Yeqing 2015	1031	147		14.3	[12.2; 16.5]	0.6%
Lei Qiong 2014	5498	726		13.2	[12.3; 14.1]	0.6%
Zhu Hui 2018	596	76		12.8	[10.2; 15.7]	0.6%
Zhang Yihui 2017	11006	1359		12.3	[11.7; 13.0]	0.6%
Zhang Jingjing 2013	1270	154		12.1	[10.4; 14.0]	0.6%
Huang Huafei 2018	33958	4090		12.0	[11.7; 12.4]	0.7%
Xiong Jiangjun 2017	3190	378		11.8	[10.7; 13.0]	0.6%
Qin Jiabi 2017	5639	661		11.7	[10.9; 12.6]	0.6%
Zhao Quming 2019	122765	13995		11.4	[11.2; 11.6]	0.7%
Zhu Li 2015	159334	17495		11.0	[10.8; 11.1]	0.7%
Wang Renhong 2018	33804	3682		10.9	[10.6; 11.2]	0.7%
Yan Yan 2017	1683	183		10.9	[9.4; 12.5]	0.6%
Liu Weiliang 2016	6000	650		10.8	[10.1; 11.6]	0.6%
Fan Lichun 2013	89854	9705		10.8	[10.6; 11.0]	0.7%
Ji Deyong 2015	6151	664		10.8	[10.0; 11.6]	0.6%
Liu Lei 2012	702	74		10.5	[8.4; 13.1]	0.6%
Hou Xiaojing 2018	600	62		10.3	[8.0; 13.1]	0.6%
Peng Tingting 2015	4804	494		10.3	[9.4; 11.2]	0.6%
Zhao Xiaoli 2018	786	80		10.2	[8.2; 12.5]	0.6%
Zhang Xiaobin 2015	10880	1100		10.1	[9.5; 10.7]	0.6%
Yu Yanhua 2012	2436	244		10.0	[8.9; 11.3]	0.6%
Liu Xiaohui 2016	10179	1019		10.0	[9.4; 10.6]	0.6%
Xu Fengdan 2019	138247	13701		9.9	[9.8; 10.1]	0.7%
Liu Xiyong 2014	13381	1325		9.9	[9.4; 10.4]	0.6%
Zhu Yan 2012	106078	10498		9.9	[9.7; 10.1]	0.7%
Zhang Xiaosong 2016	111095	10986		9.9	[9.7; 10.1]	0.7%
Xu Xiaoying 2015	6181	608		9.8	[9.1; 10.6]	0.6%
MCT for RDS 2016	62880	6017		9.6	[9.3; 9.8]	0.7%
Han Yingying 2018	6693	638		9.5	[8.8; 10.3]	0.6%
Chen Chang 2019	75590	7179		9.5	[9.3; 9.7]	0.7%
Sun Pengfei 2017	67718	6213		9.2	[9.0; 9.4]	0.7%
Jiang Fang 2016	14544	1328		9.1	[8.7; 9.6]	0.6%
Zhou Xin 2014	11302	1030		9.1	[8.6; 9.7]	0.6%
Liu Xijuan 2019	19800	1800		9.1	[8.7; 9.5]	0.6%
Li Pengcheng 2017	29712	2662		9.0	[8.6; 9.3]	0.6%
Yu Honghui 2018	797	71		8.9	[7.0; 11.1]	0.6%
Tian Qing 2013	34572	3045		8.8	[8.5; 9.1]	0.6%
Zhong Shilin 2016	19203	1688		8.8	[8.4; 9.2]	0.6%
Ke Li 2018	3684	323		8.8	[7.9; 9.7]	0.6%
Shen Chongrong 2015	1564	136		8.7	[7.3; 10.2]	0.6%
Fernando C. Barros 2015	6510	547		8.4	[7.7; 9.1]	0.6%
Zhao Qianqian 2016	11142	930		8.3	[7.8; 8.9]	0.6%
Zhou J 2014	1953	159		8.1	[7.0; 9.4]	0.6%
Dong Renjing 2017	14243	1149		8.1	[7.6; 8.5]	0.6%
Feng Fangfang 2013	1400	110		7.9	[6.5; 9.4]	0.6%
Zhuang Xun 2017	36755	2875		7.8	[7.5; 8.1]	0.6%
Liu Yajun 2017	65173	5092		7.8	[7.6; 8.0]	0.7%
Sun Jianhua 2013	2370	184		7.8	[6.7; 8.9]	0.6%
Yan Junmei 2018	60730	4698		7.7	[7.5; 8.0]	0.7%
Zeng Lixiang 2017	34082	2616		7.7	[7.4; 8.0]	0.6%
Tan Jing 2017	21942	1684		7.7	[7.3; 8.0]	0.6%
Guo Tongjun 2017	55654	4212		7.6	[7.3; 7.8]	0.7%
Chen Ping 2015	1958	148		7.6	[6.4; 8.8]	0.6%
Liu Lu 2015	2973	223		7.5	[6.6; 8.5]	0.6%
Huang Xin 2018	16780	1249		7.4	[7.1; 7.9]	0.6%
Tang Wen 2017	3915965	289381		7.4	[7.4; 7.4]	0.7%
Xu Rong 2016	157187	11530		7.3	[7.2; 7.5]	0.7%
Lu Rongxian 2012	3316	243		7.3	[6.5; 8.3]	0.6%
Wang Xiaojie 2018	6011	439		7.3	[6.7; 8.0]	0.6%
Wang Shiwen 2017	10933	793		7.3	[6.8; 7.8]	0.6%
Yi Lilan 2013	5539	399		7.2	[6.5; 7.9]	0.6%
Du Huijuan 2016	10644	764		7.2	[6.7; 7.7]	0.6%
Guo Tongjun 2018	1020471	73240		7.2	[7.1; 7.2]	0.7%
Ding Jie 2016	642	46		7.2	[5.3; 9.4]	0.5%
Luo Xiaolin 2015	111767	7872		7.0	[6.9; 7.2]	0.7%
Liu Yiwen 2018	993	69		6.9	[5.4; 8.7]	0.6%
Kang Huixian 2017	1126	78		6.9	[5.5; 8.6]	0.6%
Yuan Xiaosong 2019	11576	797		6.9	[6.4; 7.4]	0.6%
Xue Qinqin 2016	37443	2541		6.8	[6.5; 7.0]	0.6%
Liu Jingli 2013	3141	211		6.7	[5.9; 7.6]	0.6%
Xu Qin 2018	4832887	324207		6.7	[6.7; 6.7]	0.7%
Ren Dafeng 2013	4360	292		6.7	[6.0; 7.5]	0.6%
Shan Xiaoyi 2014	22507	1496		6.6	[6.3; 7.0]	0.6%
Yu Huiting 2016	191100	12513		6.5	[6.4; 6.7]	0.7%
Yan Huina 2019	3688	241		6.5	[5.8; 7.4]	0.6%
Wang Chengshu 2013	4090	261		6.4	[5.7; 7.2]	0.6%
Li Shanshan 2017	1547	98		6.3	[5.2; 7.7]	0.6%
Xin Qianqian 2017	21994	1392		6.3	[6.0; 6.7]	0.6%
Guan Tianjia 2019	82000	5166		6.3	[6.1; 6.5]	0.7%
Guo Yong 2014	54575	3384		6.2	[6.0; 6.4]	0.6%
Ye Lin 2018	24246	1501		6.2	[5.9; 6.5]	0.6%
Han Shujing 2016	37573	2294		6.1	[5.9; 6.4]	0.6%
Li Changchang 2018	587829	34946		5.9	[5.9; 6.0]	0.7%
Yang M. 2019	4340	252		5.8	[5.1; 6.5]	0.6%
Zhang Dandan 2016	11020	630		5.7	[5.3; 6.2]	0.6%
Yu Rong 2015	10678	588		5.5	[5.1; 6.0]	0.6%
Zhao Jinqi 2018	1366377	74994		5.5	[5.5; 5.5]	0.7%
Su Weijuan 2019	62852	3350		5.3	[5.2; 5.5]	0.6%
Zheng Feng 2013	5472	290		5.3	[4.7; 5.9]	0.6%
Xia Min 2017	10868	571		5.3	[4.8; 5.7]	0.6%
Xu Jing 2018	4552	239		5.3	[4.6; 5.9]	0.6%
Wang Chen 2017	14970	785		5.2	[4.9; 5.6]	0.6%
Li Xiangyu 2018	10635	557		5.2	[4.8; 5.7]	0.6%
Wei Liangjia 2019	12780	668		5.2	[4.8; 5.6]	0.6%
Ye Juan 2017	2463	125		5.1	[4.2; 6.0]	0.6%
Xu Hairong 2013	1785	90		5.0	[4.1; 6.2]	0.6%
Lin Xianhua 2019	49612	2494		5.0	[4.8; 5.2]	0.6%
Hu Ronghua 2017	317463	15889		5.0	[4.9; 5.1]	0.7%
Bo Sun 2015	383976	19198		5.0	[4.9; 5.1]	0.7%
Guo Pi 2019	1098600	54521		5.0	[4.9; 5.0]	0.7%
Liu Tao 2019	21891	1080		4.9	[4.7; 5.2]	0.6%
He Jianrong 2018	9044	444		4.9	[4.5; 5.4]	0.6%
Yang Wenjia 2017	979	48		4.9	[3.6; 6.4]	0.5%
Liu Shiping 2017	882	43		4.9	[3.6; 6.5]	0.5%
Xiao Yue 2018	1569	76		4.8	[3.8; 6.0]	0.6%
Li Guijuan 2013	4214	202		4.8	[4.2; 5.5]	0.6%
Dai Xiaowei 2019	1377	66		4.8	[3.7; 6.1]	0.6%
Wang Chen 2018	21577	1027		4.8	[4.5; 5.1]	0.6%
Tang Jiangbing 2018	79850	3778		4.7	[4.6; 4.9]	0.6%
Li Run 2017	688	32		4.7	[3.2; 6.5]	0.5%
Wang Yaocheng 2018	120819	5532		4.6	[4.5; 4.7]	0.7%
Pan Lei 2015	17545	803		4.6	[4.3; 4.9]	0.6%
Hu Jie 2019	16345	721		4.4	[4.1; 4.7]	0.6%
Xiao Qingyang 2018	132783	5856		4.4	[4.3; 4.5]	0.7%
He Jifei 2014	8567	366		4.3	[3.9; 4.7]	0.6%
Xue Xiaoping 2015	50871	2163		4.3	[4.1; 4.4]	0.6%
Xu Xin 2014	6270	265		4.2	[3.7; 4.8]	0.6%
Yang Qing 2012	9741	409		4.2	[3.8; 4.6]	0.6%
Long Xinzhi 2016	2000	83		4.2	[3.3; 5.1]	0.6%
Guo Yawei 2013	6270	259		4.1	[3.7; 4.7]	0.6%
Zhang Cuihong 2015	14196	586		4.1	[3.8; 4.5]	0.6%
Zhu Beibei 2019	3273	134		4.1	[3.4; 4.8]	0.6%
Ding Haiyan 2018	18276	748		4.1	[3.8; 4.4]	0.6%
Wang Fen 2016	24145	986		4.1	[3.8; 4.3]	0.6%
Ma Haihui 2019	34726	1418		4.1	[3.9; 4.3]	0.6%
Zhou Miao 2015	7171	289		4.0	[3.6; 4.5]	0.6%
Tian Jiarong 2018	10122	404		4.0	[3.6; 4.4]	0.6%
Zhao Yanjie 2017	34022	1339		3.9	[3.7; 4.1]	0.6%
Wang H. 2018	119454	4643		3.9	[3.8; 4.0]	0.7%
Hu Chengyang 2018	16983	656		3.9	[3.6; 4.2]	0.6%
Zheng Wei 2018	10366	400		3.9	[3.5; 4.2]	0.6%
Fei Lixiao 2014	19120	727		3.8	[3.5; 4.1]	0.6%
Fang Haiqin 2017	10241	369		3.6	[3.3; 4.0]	0.6%
Yang Jin 2018	2320	82		3.5	[2.8; 4.4]	0.6%
Wen Tingyuan 2015	13776	485		3.5	[3.2; 3.8]	0.6%
Jia Xiaomin 2015	2729	96		3.5	[2.9; 4.3]	0.6%
Chen Liangmiao 2014	8012	281		3.5	[3.1; 3.9]	0.6%
Huang Aiqun 2018	40152	1331		3.3	[3.1; 3.5]	0.6%
Fan Xiaojing 2017	701	23		3.3	[2.1; 4.9]	0.4%
Fang Jing 2019	1028	33		3.2	[2.2; 4.5]	0.5%
Li Yanhua 2014	592	19		3.2	[1.9; 5.0]	0.4%
Li Minmin 2019	28841	894		3.1	[2.9; 3.3]	0.6%
Hu Yan 2016	5242	160		3.1	[2.6; 3.6]	0.6%
Jin Wenyuan 2016	934	27		2.9	[1.9; 4.2]	0.5%
Zhao Xiang 2018	1300	37		2.8	[2.0; 3.9]	0.5%
Guo Tongjun 2017	244305	6709		2.7	[2.7; 2.8]	0.7%
Guo Yanwei 2016	4966	131		2.6	[2.2; 3.1]	0.6%
Wang Jing 2014	4042	103		2.5	[2.1; 3.1]	0.6%
Jiang Yan 2016	5000	120		2.4	[2.0; 2.9]	0.6%
Wang Mei 2013	600	13		2.2	[1.2; 3.7]	0.3%
Wang Feixue 2019	520695	8151		1.6	[1.5; 1.6]	0.7%
**Random effects model**	**17900020**	**1170288**		**6.6**	**[6.3; 6.9]**	**100.0%**

Heterogeneity: $I^2 = 99.8\%$, $\chi^2_{160} = 86967.5$ ($P < 0.001$)

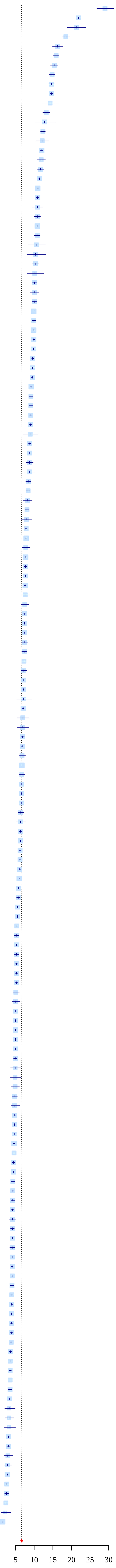

eFigure1. Forest plot of all the included studies (n=161)

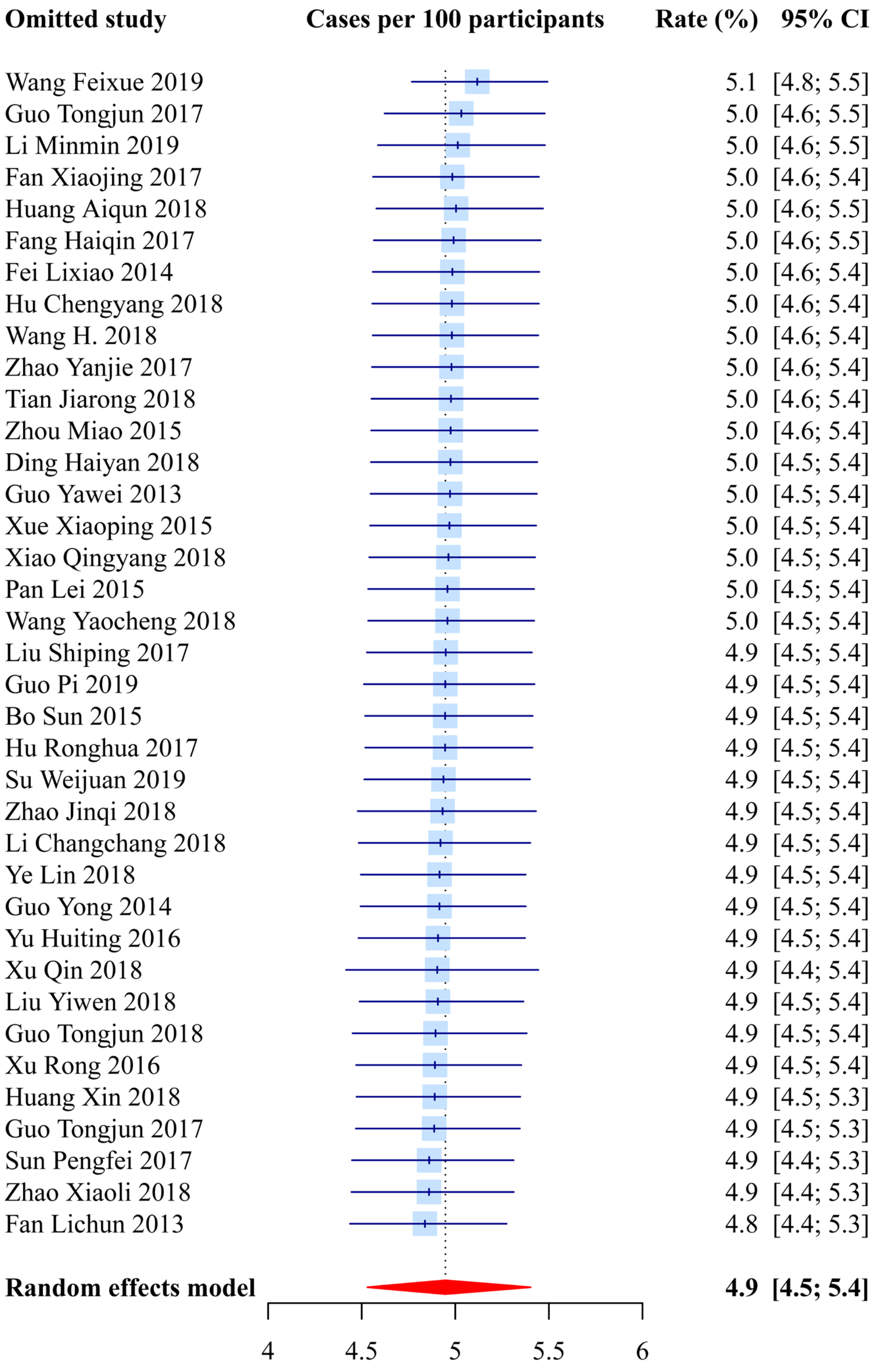

eFigure2. Sensitivity analysis within population-based studies (n=37)
